# Genetics and Regulatory Impact of Alternative Polyadenylation in Human B-Lymphoblastoid Cells

**DOI:** 10.1371/journal.pgen.1002882

**Published:** 2012-08-16

**Authors:** Oh Kyu Yoon, Tiffany Y. Hsu, Joo Hyun Im, Rachel B. Brem

**Affiliations:** Department of Molecular and Cell Biology, University of California Berkeley, Berkeley, California, United States of America; University of California San Diego, United States of America

## Abstract

Gene expression varies widely between individuals of a population, and regulatory change can underlie phenotypes of evolutionary and biomedical relevance. A key question in the field is how DNA sequence variants impact gene expression, with most mechanistic studies to date focused on the effects of genetic change on regulatory regions upstream of protein-coding sequence. By contrast, the role of RNA 3′-end processing in regulatory variation remains largely unknown, owing in part to the challenge of identifying functional elements in 3′ untranslated regions. In this work, we conducted a genomic survey of transcript ends in lymphoblastoid cells from genetically distinct human individuals. Our analysis mapped the *cis*-regulatory architecture of 3′ gene ends, finding that transcript end positions did not fall randomly in untranslated regions, but rather preferentially flanked the locations of 3′ regulatory elements, including miRNA sites. The usage of these transcript length forms and motifs varied across human individuals, and polymorphisms in polyadenylation signals and other 3′ motifs were significant predictors of expression levels of the genes in which they lay. Independent single-gene experiments confirmed the effects of polyadenylation variants on steady-state expression of their respective genes, and validated the regulatory function of 3′ *cis*-regulatory sequence elements that mediated expression of these distinct RNA length forms. Focusing on the immune regulator IRF5, we established the effect of natural variation in RNA 3′-end processing on regulatory response to antigen stimulation. Our results underscore the importance of two mechanisms at play in the genetics of 3′-end variation: the usage of distinct 3′-end processing signals and the effects of 3′ sequence elements that determine transcript fate. Our findings suggest that the strategy of integrating observed 3′-end positions with inferred 3′ regulatory motifs will prove to be a critical tool in continued efforts to interpret human genome variation.

## Author Summary

Messenger RNAs carry the instructions necessary to synthesize proteins that do work for the cell. Extending beyond the protein-coding sequence of a given mRNA is an additional stretch of sequence, harboring signals that govern how much protein is made and how long the mRNA remains in the cell before it is broken down. The incorporation of this end region into mature mRNA is itself subject to change; for the vast majority of human genes, how and why cells use different mRNA ends remains largely unknown. In this work, we surveyed mRNA ends from ∼10,000 genes in immune cells from genetically distinct human individuals. We found that mRNA end positions were not randomly distributed, but rather preferentially flanked the locations of regulatory signals that govern mRNA fate. The usage of these mRNA length forms and regulatory elements varied across individuals and could be dissected molecularly. Our results uncover key mechanisms and regulatory effects of transcript end processing, particularly as these are perturbed by genetic differences between humans.

## Introduction

Naturally occurring genetic differences in gene regulation within populations underlie phenotypes of evolutionary and biomedical interest [1]–[3] and can serve as the basis for inference of regulatory networks [4], [5]. A key problem in the field is understanding the molecular mechanisms by which DNA sequence variants give rise to expression change. Recent work has emphasized the importance of sequence differences in regions upstream of gene loci that harbor *cis*-acting determinants of transcription factor binding [6]–[10] and chromatin architecture [11]–[13]. Much less is known about the role of 3′-end regulation as a determinant of expression variation between individuals. Alternative polyadenylation represents a major regulatory strategy in the human genome, with analysis across tissue types detecting multiple 3′ UTR forms of over half of all human genes [14]. Detailed genetic studies have implicated polymorphisms affecting transcript termination in both Mendelian and complex human disease [15]–[20]. Genomic analyses have hinted at a broader role for genetic differences in RNA 3′-end processing as a driver of expression variation [6], [8], [21], [22], but the prevalence and the mechanisms of these changes are incompletely understood.

Progress in dissecting the genetics of 3′-end processing has been limited in part by fundamental questions about the regulatory information encoded in 3′ UTRs. Single-gene studies have made clear that, in addition to its interplay with exonic splicing [23], [24], RNA 3′-end processing can dictate the extent of 3′ UTR sequence incorporated into mature transcripts that governs half-life, translation, and localization [25]–[29]; the efficiency of transcription termination itself can also influence steady-state expression level of a given length form [30]–[34]. In general, however, identifying the regulatory elements that underlie relationships between 3′ UTR sequence and gene expression remains a primary challenge, and for the majority of human genes, the regulatory impact of alternative polyadenylation is unknown. Likewise, the search for molecular players underlying *cis*-regulation of 3′-end processing at individual gene loci [23] and genome-scale regulation of 3′-end processing in *trans* [14], [29], [35]–[40] is an area of active research.

A complete understanding of the genetics of alternative polyadenylation will require maps of transcript end site usage and 3′ *cis*-regulatory elements, and analysis strategies to integrate the data. Recently developed short-read sequencing methods for transcript ends [40]–[46] have enabled the possibility of quantitative studies of the regulatory architecture of transcript end forms on a genomic scale. In this work, we set out to investigate mechanisms by which alternative polyadenylation impacts gene expression and its variation across genetically distinct human individuals. We used 3′-end RNA-seq [42] to maximize the genomic coverage and precision of transcript end positions, and to measure quantitative expression levels of transcript forms. The results shed light on the architecture of transcript ends and regulatory elements in human 3′ UTRs and the principles of genetic variation in 3′ length form usage.

## Results

### Surveying transcript ends by 3′-end RNA–seq

**Figure 1:**
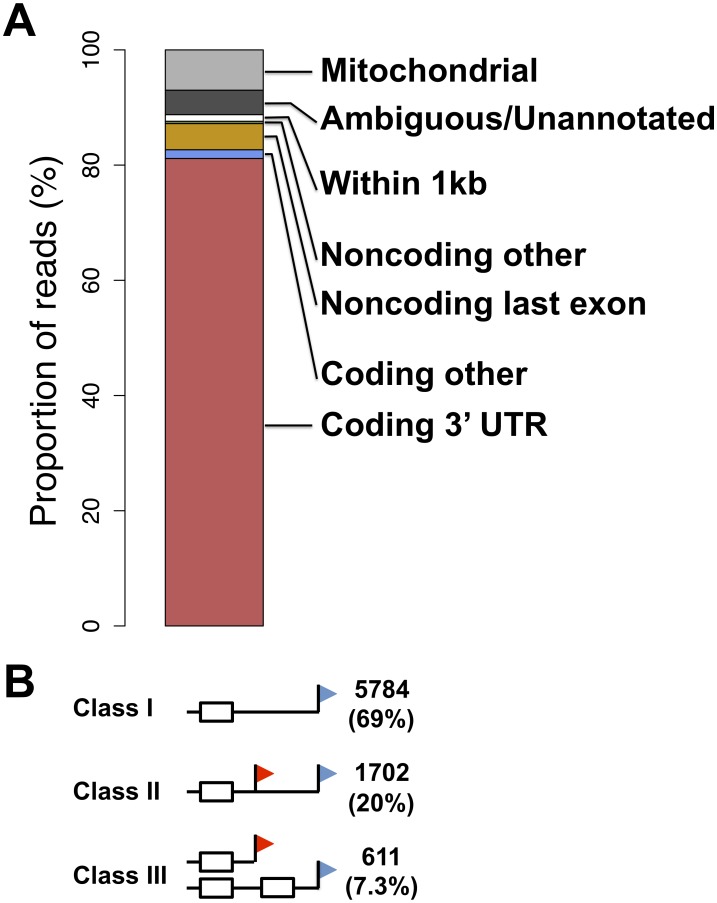
A survey of transcript ends and alternative polyadenylation. (A) Shown is the agreement between genomic feature boundaries from the UC Santa Cruz human genome annotation [63] and 3′-end cleavage positions from all samples and replicates of 3′-end RNA-seq applied to B-lymphoblastoid cell lines. (B) White boxes represent exons, black horizontal lines represent noncoding sequence, and red and blue flags represent proximal and distal transcript end positions, respectively. Counts at right represent numbers of coding genes expressed in lymphoblastoid cells showing evidence for the indicated transcript end form patterns, with proportions in parentheses.

To survey the 3′ ends of transcripts in human B-lymphoblastoid cells, we isolated RNA from cell lines derived from six human individuals and subjected each replicate of each sample to 3′-end RNA-seq, which sequences polyadenylated transcript ends on a genomic scale [42] with strong and significant reproducibility (Figure S1). Our analysis pipeline, described in Materials and Methods, filtered out likely products of mispriming from A-rich genomic regions [47] and categorized mapping reads in terms of well-defined peaks or dispersed regions of reads with no peak structure (Table S1). We considered the former to represent the strongest candidates for stable, functional transcript end forms, and focused on these for in-depth analysis, taking the last mapped base of each read as the likely site at which the nascent RNA was cleaved from the processing polymerase and polyadenylated [24]. Across such transcript ends mapping to nuclear-encoded loci, the vast majority of reads (87%) originated from 3′ UTRs of coding genes (Figure 1A and Table S1); 80% of transcript ends were consistent with the 3′ ends of previously annotated length forms (Tables S1 and S2 and Figure S2). Within 40 base pairs of the inferred cleavage position, most transcript end forms harbored a polyadenylation signal sequence: either the canonical A(A/U)UAAA [23] or a close variant, or an A-rich stretch [48]–[50] (Figure S3A). Reporter assays confirmed the regulatory importance of A-rich stretches upstream of the inferred cleavage positions in CPSF1 and WDR18, whose 3′ UTRs lacked canonical polyadenylation signal motifs (Figure S3C).

We set out to analyze our data set of 3′ transcript ends with respect to alternative polyadenylation, focusing on a maximum of two abundant, distinguishable transcript forms in a given gene. We considered three patterns of transcript end usage [30]: class I, indicating genes with a single transcript form terminating in an annotated 3′ UTR; class II, genes with alternative polyadenylation in the same annotated 3′ UTR; and class III, genes in which the two alternative polyadenylation forms differed in their composition of coding sequence (Figure 1B). We also observed a small fraction of genes with a single transcript form terminating inside annotated coding exons or introns (Table S3). The breakdown of transcript forms into these classes revealed alternative polyadenylation reaching our threshold of detection in ∼30% of genes (Figure 1B). Across these genes, we observed the expected enrichment of the A(A/U)UAAA polyadenylation signal motif in the distal relative to proximal polyadenylation signals (Figure S3A), correlating with the higher expression levels of the long transcript forms [30], [51]. The lower-abundance short transcript forms were more likely to harbor an A-rich stretch or no recognizable polyadenylation signal motif upstream of the inferred cleavage position (Figure S3B), lending credence to the notion that the latter regions represent weak recognition sites for the 3′-end processing machinery [48].

### Interplay of 3′ cis-regulatory elements with alternative polyadenylation

**Figure 2:**
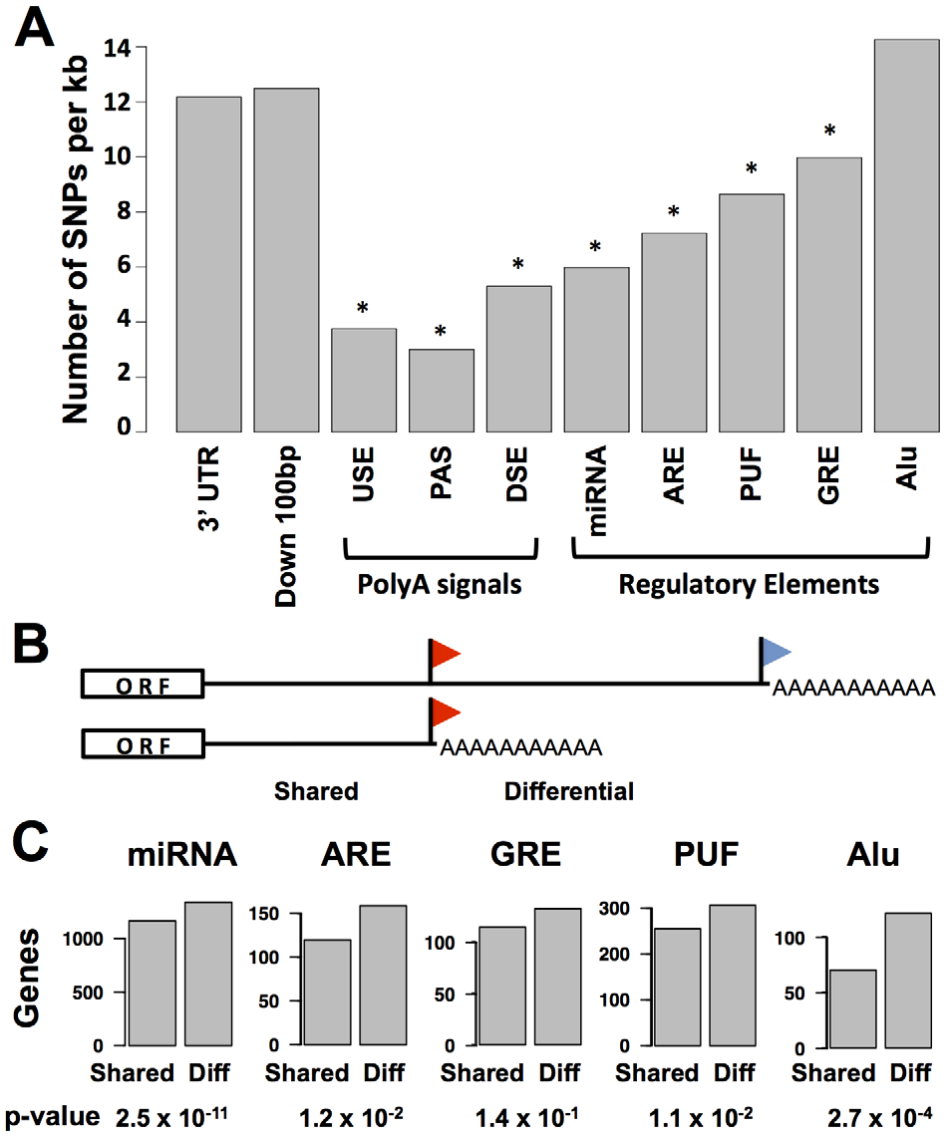
Conservation and positioning of regulatory elements in 3′ untranslated regions. (A) Each bar represents all sequence regions across genes in classes I, II, and III (see Figure 1B) meeting bioinformatic criteria for the indicated 3′ region or motif as described in Materials and Methods. For each set of regions, the *y*-axis reports the density of single nucleotide polymorphisms across human genome sequences from the 1000 Genomes project (www.1000genomes.org). Asterisks represent motifs with a lower density of variants than that predicted by a 3′ UTR background model, as reported in Table S4. Down 100 bp, 100 base-pair windows downstream of 3′ cleavage positions; USE, U-rich upstream transcription termination element; PAS, polyadenylation signal; DSE, U/G-rich downstream transcription termination element; miRNA, microRNA binding site; ARE, A/U rich element; GRE, G/U-rich element; PUF, binding site for the Pumilio family of proteins; Alu, Alu transposable elements. (B) Schematic illustrating shared and differential regions for a class II gene; symbols are as in Figure 1B. (C) Each panel reports analysis of all class II genes (see Figure 1B); the *y*-axis shows the number of genes with matches to the indicated motif in the shared or differential 3′ untranslated sequence regions across the gene set. *p*-values from a two-sided Fisher's exact test comparing the numbers of genes with motifs in shared and differential regions are shown below.

We sought to harness our data set of transcript ends to investigate regulatory elements governing translation and transcript half-life, and their relationship to alternative 3′ transcript forms. For this purpose, we applied a motif-search strategy to identify putative microRNA binding sites, A/U rich elements (AREs), G/U-rich elements, binding sites for the Pumilio family of proteins, and Alu transposable elements in 3′ UTRs. We tabulated rates of sequence variation across human populations and observed marked conservation of most regulatory element motifs relative to the background level of 3′ UTR polymorphism (Figure 2A), reflecting a history of purifying selection on these putatively functional regulatory sequences [52]–[54].

We expected that the regulatory logic of alternative polyadenylation would be intimately connected with sequence determinants of transcript half-life or translation in 3′ UTRs. For a given gene subject to alternative polyadenylation, we referred to the region of the 3′ UTR upstream of the proximal cleavage site as “shared” among the alternative polyadenylation length forms, and the span of the 3′ UTR in between the proximal and distal cleavage sites as the “differential” region (Figure 2B). We hypothesized that, if alternative polyadenylation often acted to tune the exposure of *cis*-regulatory motifs in 3′ UTR sequences, these motifs would preferentially be positioned in differential regions. To test this, we tabulated the positions of each type of regulatory element across our set of alternatively polyadenylated genes in class II. Significance testing revealed a significant enrichment of genes with motifs in the differential regions of 3′ UTRs relative to those with motifs in shared regions (Figure 2C). Analyzing the annotations of genes in these sets, we observed a preponderance of genes with immune-related functions among those with *cis*-regulatory motifs in the differential regions of 3′ UTRs, while motifs in shared regions of 3′ UTRs were largely detected among genes with housekeeping roles (Table S5). Thus, for genes carrying out immune processes in B-lymphoblastoid cells, the choice between long and short transcript end forms often exposes or eliminates regulatory information in 3′ UTRs, highlighting the importance of 3′-end processing in the control of gene expression levels for specialized cell functions.

### Genetic variation in 3′-end processing and the role of polyadenylation signals

To investigate the genetics of RNA 3′-end processing, we first assessed the contribution of genetic differences, relative to experimental and environmental error, to variation of transcript 3′-end positions across the six genotypes of lymphoblastoid cells in our study. For this purpose, we calculated the heritability of length form abundance for each gene, finding 194 coding genes at which the abundances of transcript length forms differed reproducibly (heritability>0.6) across human samples (Table S6). To begin to dissect the molecular basis for natural genetic variation in 3′-end usage at these loci, we considered the potential role for DNA sequence differences at polyadenylation signals, the primary determinants of transcript cleavage and polyadenylation. We reasoned that although such variants were rare in the human population (Figure 2A), some could underlie differences between human individuals in 3′-end length form usage. Consistent with this prediction, genes with highly heritable transcript end positions harbored a single nucleotide polymorphism in polyadenylation signal motif sequence much more often than did the average gene (2% of polyadenylation signals at genes with heritable transcript ends, compared to 0.3% genome-wide; Fisher's exact *p* = 0.004; Table S6).

**Figure 3:**
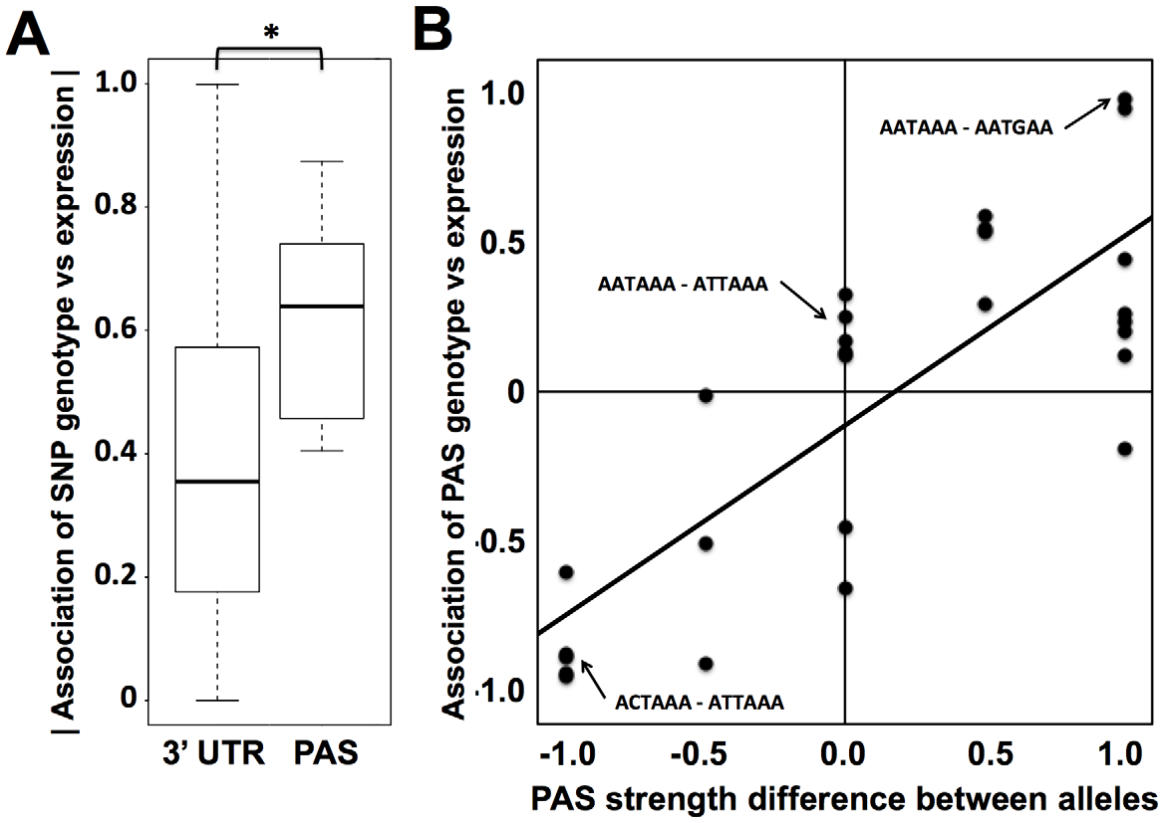
Sequence variation in polyadenylation signals as a predictor of expression change across human individuals. (A) Each column represents analyses of all DNA sequence variants in 3′ UTRs (3′ UTR) or variants in inferred polyadenylation signals (PAS). For each variant, analysis comprised a linear regression across genetically distinct individuals between two sets of measurements: inheritance at the locus, scored in terms of the number of major alleles in the diploid genotype, and expression of the gene in which it lay. The *y*-axis reports a distribution of the absolute values of Pearson correlation coefficients from such linear regressions across all variants in the indicated regions (Materials and Methods). In each distribution, the median is reported as a thick horizontal line, the 25% quantile is shown as a box, and the extremes are shown as thin horizontal bars. The asterisk represents a comparison significant at *p*<0.01 by a one-sided Wilcoxon test. (B) Each point represents analysis of biallelic sequence variants in polyadenylation signals and expression of the genes in which they lay. For each allele of each variant, the polyadenylation signal was assigned a score from 1 to 0 representing the strength of the match to the canonical polyadenylation signal motif (Materials and Methods); the *x*-axis reports the difference between such scores of the two alleles, with positive values indicating a stronger match to the canonical motif by the major allele. The *y*-axis reports the results of a linear regression between the polyadenylation signal variant and expression as in (A), with positive values representing increased expression associated with the major allele. The black diagonal line is a linear regression fit. Example polyadenylation signal variants exhibiting each score value are shown in small text, with the major allele followed by the minor allele.

We hypothesized that naturally occurring genetic variation in RNA 3′-end processing would prove to underlie changes across individuals in steady-state levels of gene expression. To test this, we analyzed the genomic relationship between biallelic single-nucleotide polymorphisms in polyadenylation signals and gene expression differences across our set of lymphoblastoid cell lines from distinct human genotypes. We used a standard regression test of genetic association to evaluate genotype at each such sequence variant as a predictor of expression of the gene in which it lay. The results revealed a significant enrichment of association with expression for variants in polyadenylation signals, relative to the background signal from 3′ UTRs as a whole (Figure 3A). We expected that for a given such variant, the allele conferring a closer match to the canonical polyadenylation signal motif would confer more robust transcription termination, and thus more abundant steady-state levels of transcription, than would the allele weakening the match to the canonical motif. To quantify this effect, we scored each allele in each variant polyadenylation signal with respect to agreement with the canonical motif, and at each variant position, we calculated the difference in scores between alleles. This score difference was a strong predictor of the effect of a given polyadenylation signal variant on steady-state expression levels (Figure 3B), with a departure from the polyadenylation signal motif associated with a drop in expression as predicted.

**Figure 4:**
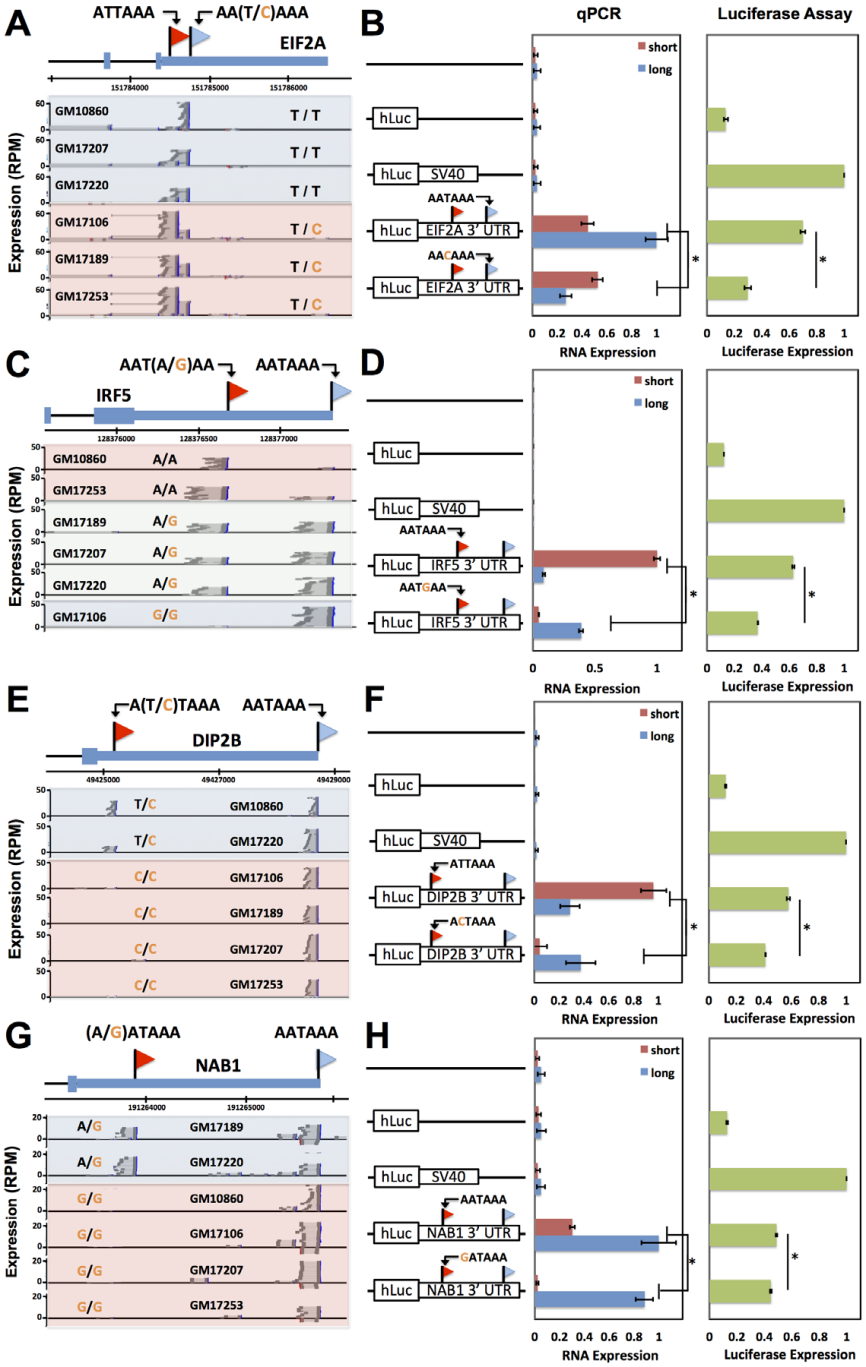
Natural genetic variation in alternative polyadenylation. Each row reports characterization of one gene at which 3′ end usage varied across human B-lymphoblastoid cell lines from genetically distinct individuals. (A), (C), (E), (G) Each panel reports 3′-end RNA-seq data for one gene. The top cartoon shows exons (short boxes), 3′ untranslated regions (long bars), transcript ends (flags), and polyadenylation signals (text). Each bottom shaded section shows 3′-end RNA-seq reads(dark grey lines) and the sequences between read pairs (light grey lines) from a lymphoblastoid cell line derived from one human individual, with poly-A tails in blue. Alleles at center indicate the genotype of the indicated individual at the polymorphic polyadenylation signal sequence shown at top. (B), (D), (F), (H) Each panel reports results from a luciferase reporter (hLuc) or luciferase-negative control (unmarked horizontal line) transfected into HEK293T cells. At left, each cartoon shows schematics of reporters; each horizontal box labeled with a gene name represents the indicated 3′ untranslated region from that gene, cloned from one cell line and mutagenized to introduce a natural variant in a polyadenylation signal as shown. SV40, positive control terminator region from simian virus 40. For each reporter, center panels show measurements of abundance of one transcript length form, interrogated by quantitative PCR using primers specific to short or long transcript ends inferred from 3′-end RNA-seq; asterisks represent results significant at *p*<0.03 by a two-sided Wilcoxon test comparing the ratio of abundances of short and long forms between the indicated reporters, and error bars represent standard deviations (*n* = 4). Complementary measurements *via* Northern blot are shown in Figure S4. Right panels show luciferase protein activity; asterisks represent results significant at *p*<0.03 by a two-sided Wilcoxon test comparing the abundances between the indicated reporters, and error bars represent standard deviations (*n* = 4). For each assay and each gene, all expression values were normalized against the sample with highest expression.

To pursue on a molecular basis the impact of variation across humans in polyadenylation signals, we used our 3′-end RNA-seq data to infer the effects of single-nucleotide variants on usage of 3′ transcript forms at individual genes, and we evaluated these predictions in single-gene 3′ UTR reporter assays. For each gene, toggling natural variant alleles at one nucleotide position in the polyadenylation signal was sufficient to drive differential usage of short and long 3′ transcript forms (Figure 4). These included variants attenuating usage of 3′ forms of the translation initiation factor EIF2A and the putative DNA methylation enzyme DIP2B, as well as the expected effect of the polymorphic polyadenylation signal on usage of 3′ forms of the inflammation regulator IRF5 [15]. In each of the latter genes, the causal variant conferred significant changes in luciferase protein levels as well as usage of transcript forms (Figure 4A–F). Our set of confirmed causal variants at polyadenylation signals also included that in the transcription factor NAB1, which attenuated usage of a minor 3′ transcript form with modest effect on luciferase levels (Figure 4G, H). We conclude that sequence differences in polyadenylation signals represent a key mechanism underlying variation between humans in levels of gene expression, with genome-scale trends validated at the single-gene level.

### Sequence elements determining abundance of transcript forms and their regulatory consequences

**Figure 5:**
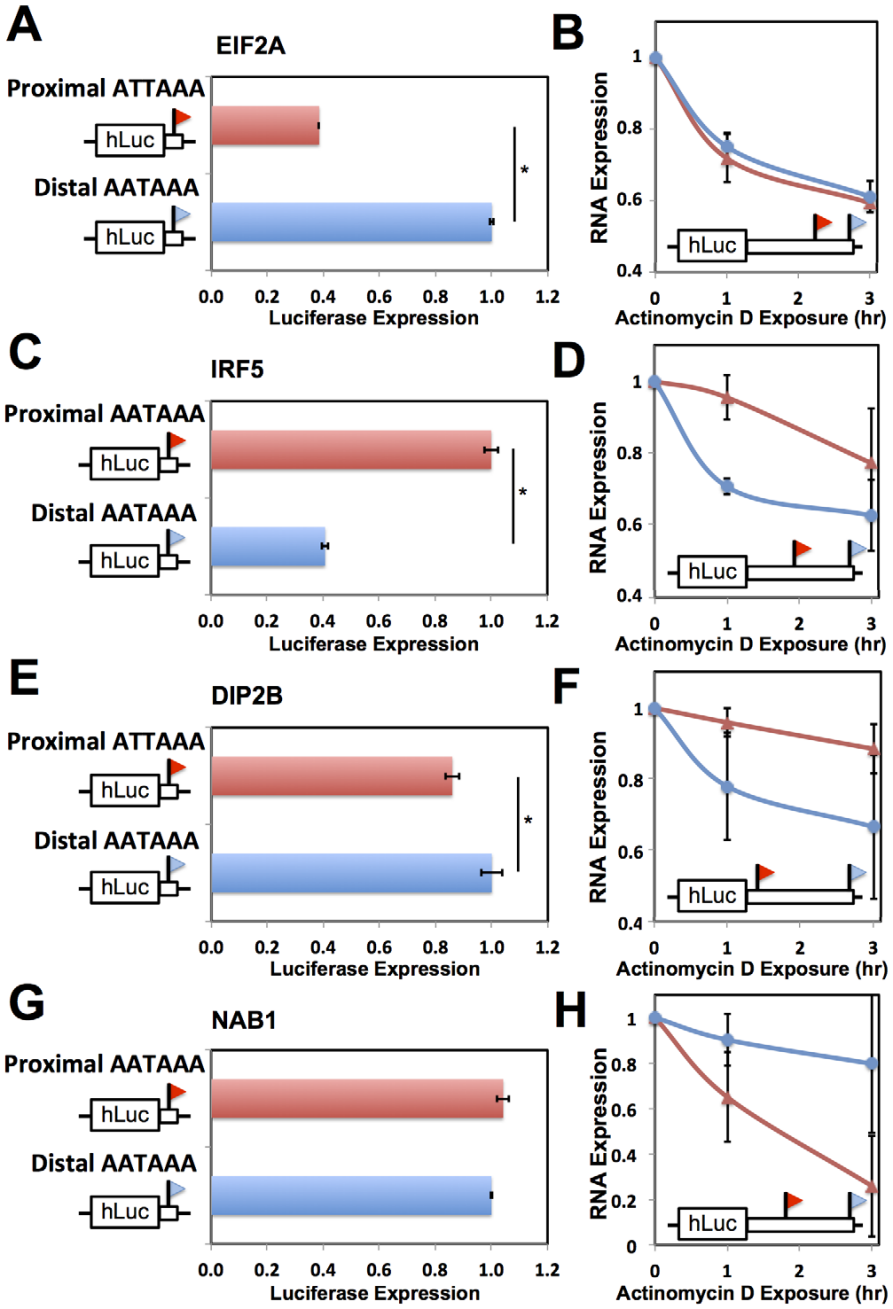
Effects of 3′ processing signals and transcript half-life on expression of short and long transcripts. Each row reports characterization of one gene at which short and long 3′ transcript forms were associated with different abundance (see Figure 4). (A), (C), (E), (G) Each panel reports results from a luciferase reporter (hLuc) incorporating minimal 3′ processing signals, and a canonical polyadenylation signal as indicated in black text at left, from the genomic regions flanking the proximal and distal ends of 3′ forms of the indicated gene (red and blue, respectively), transfected into HEK293T cells. For each reporter diagrammed at left, panels show luciferase protein activity; asterisks represent results significant at *p*<0.03 by a two-sided Wilcoxon test comparing the abundances between the indicated reporters, and error bars represent standard deviations (*n* = 4). For each assay and each gene, all expression values were normalized against the sample with higher expression. (B), (D), (F), (H) Each panel reports results from a luciferase reporter (hLuc) incorporating the complete 3′ untranslated region of the indicated gene using a human haplotype producing both short and long 3′ forms (see Figure 4), transfected into HEK293T cells which were then treated with actinomycin D. Each data point represents measurements of abundance of one transcript length form, interrogated by quantitative PCR using primers specific to proximal (red) or distal (blue) 3′ transcript ends. Error bars represent standard deviations (*n* = 2). For each transcript length form, all expression values were normalized against the sample with no actinomycin D exposure. Complementary measurements *via* Northern blot are shown in Figure S4.

We next sought to dissect the mechanisms by which natural variation in 3′-end usage impacted gene expression, using as case studies IRF5 and DIP2B, which lie in genomic regions associated with susceptibility to lupus [15] and colorectal cancer [55], respectively, as well as NAB1 and EIF2A. In RNA expression measurements using the 3′ UTR haplotype that produced both long and short transcript forms of a given gene, one form was detected at higher abundance in each case (Figure 4B, D, F, H). We hypothesized that these abundance differences between length forms could be in part the result of sequence elements that dictate transcript cleavage, polyadenylation, and termination, and in part the result of regulatory elements that affect transcript half-life. To test this, for each gene we first developed expression reporters incorporating only the regions flanking the end positions of each transcript form in turn, which we expected would include the polyadenylation signal and auxiliary sequence motifs underlying 3′-end processing of the respective form while excluding most other 3′ regulatory information. Expression measurements confirmed differences in the strength of these 3′-end processing motifs between length forms for EIF2A, IRF5, and DIP2B, in that reporters incorporating each of the two 3′-end sequences from a given gene exhibited up to 2.5-fold differences in expression (Figure 5A, C, E). To assess the contribution of 3′ regulatory elements that control transcript half-life, we next measured the decay rate of each transcript length form upon addition of actinomycin D in the context of complete 3′ UTRs. Measurements of transcript stability by quantitative PCR and by Northern blot bore out this prediction, with the long transcript form showing reduced half-life relative to the short form for IRF5 [15] and DIP2B (Figure 5D, F and Figure S4B, C), and increased half-life for NAB1 (Figure 5H and Figure S4D). Analyzing these results together with the effects of natural variants in polyadenylation signals (Figure 4) indicates that for a given gene, a variant can abrogate production of a transcript form with strong sequence determinants of 3′-end processing, leaving only the less efficiently processed form and giving rise to lower total expression of the gene product, as in IRF5 and EIF2A. In addition, a variant abrogating production of a transcript form with longer half-life leaves only the less-stable form and reduces total steady-state levels of the gene product, as in IRF5 and DIP2B.

**Figure 6:**
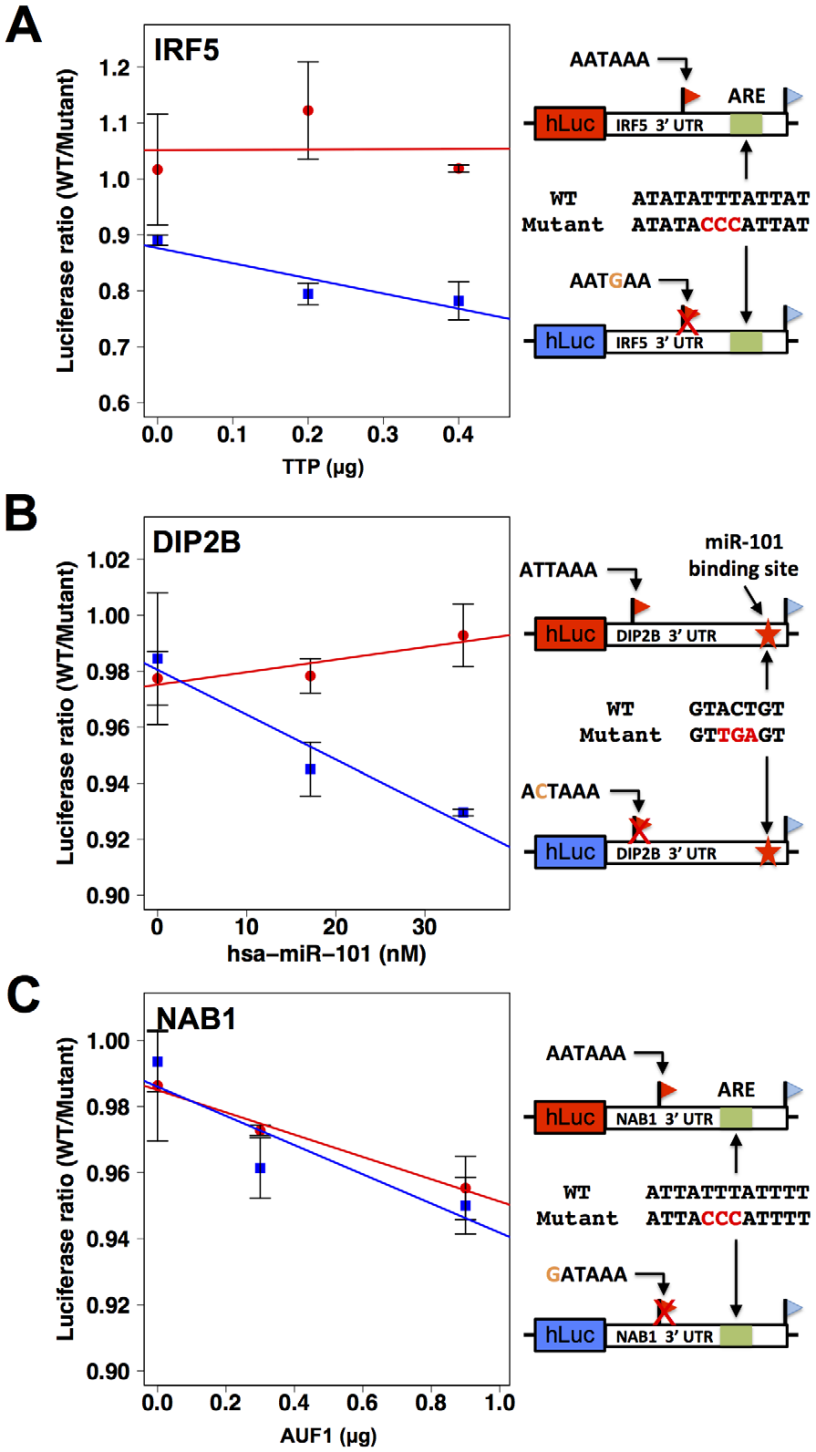
3′ sequence motifs are required for the regulatory response to *trans*-acting factors. Each row reports characterization of one gene at which short and long 3′ transcript forms were associated with different abundance (see Figure 4). At right, each cartoon shows a schematic of a luciferase reporter (hLuc) cloned upstream of the 3′ untranslated region of the indicated gene that produces two 3′ length forms (flags) and bears an inferred regulatory element as indicated (green box or red star) in the long 3′ form. For a given row, top and bottom reporters differ in the naturally occurring allele borne at the proximal polyadenylation signal (orange text): one reporter (red box) bears the allele encoding robust 3′ processing at the proximal site, and the other reporter (blue box) bears the allele eliminating the canonical polyadenylation signal at the proximal site (red X). In left panels, each color represents protein measurements from reporters with one allele at the proximal polyadenylation site, encoding either robust (red) or minimal (blue) 3′ processing. For each point, the *y*-axis reports protein abundance measurements from two versions of the reporter, one with a wild-type 3′ regulatory element (WT) and the other with an engineered mutant allele (red text at right). In each case, reporters were transfected into HEK293T cells expressing an exogenous copy of an RNA-binding regulator as indicated on the *x*-axis. Error bars represent standard deviations (*n* = 2). (A) IRF5, (B) DIP2B, (C) NAB1.

As a further investigation of the determinants of abundance of long and short transcript forms for genes subject to natural genetic change in 3′-end processing, we analyzed the role of 3′ regulatory elements in such genes in relation to *trans*-acting regulatory factors. We identified a candidate ARE in the differential region of the 3′ UTR of IRF5, *i.e.* between the positions of alternative 3′ ends observed in our 3′-end RNA-seq; a candidate ARE in the differential region of NAB1; and a candidate binding site for the miRNA miR-101 in the differential region of DIP2B (Figure 6). To assess the functional relevance of these motifs, we applied a mutagenesis strategy using 3′ UTR reporter constructs for each gene, as above distinguishing between the 3′ UTR haplotype that produced both long and short transcript forms and the haplotype producing only the long form (Figure 4D, F, H). For each inferred *cis*-regulatory element, we assayed the regulatory response of 3′ UTR reporters to the *trans*-acting factor predicted to mediate its repressive effect: the ARE-binding proteins TTP and AUF1 for IRF5 and NAB1, respectively, and a mimic of miR-101 for DIP2B. In each case, expression measurements from mutagenized reporter constructs established the respective sequence element as necessary for full repression of the long form of its host UTR (Figure 6), validating our motif inferences. Among experiments that used haplotypes producing both short and long 3′ forms of the respective UTRs, motifs in the differential region were only necessary for repression by *trans*-acting factors in the case of NAB1 (Figure 6C), for which the population of transcripts arising from this haplotype was dominated by the long form (Figure 4H). Taken together, our results illustrate the complexity of regulatory information in 3′ UTRs, as determinants of RNA 3′-end processing and transcript fate each contribute to the final expression level of the host gene. We conclude that, for these case studies, integrating predicted regulatory motifs with knowledge of transcript end positions is essential in the effort to relate genotype to gene expression.

**Figure 7:**
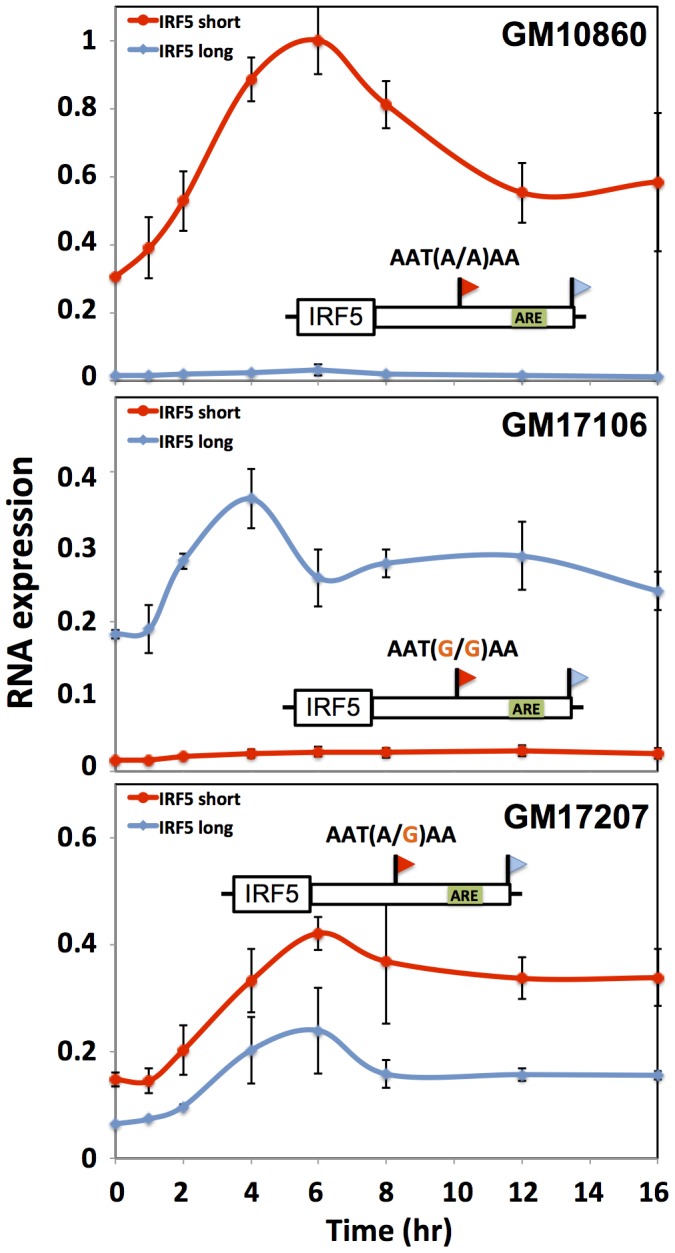
Genetic variation in IRF5 alternative polyadenylation and recovery of steady-state expression after induction. Each data point represents IRF5 expression in B-lymphoblastoid cells measured by quantitative PCR after exposure to lipopolysaccharide (5 µg/mL). Each line style represents one 3′ length form of IRF5 mRNA. Each panel represents a cell line from a genetically distinct individual as indicated at top right, whose biallelic genotype at the proximal polyadenylation signal which encodes robust (short) or minimal (long) 3′ processing is shown above the schematic of the IRF5 gene; an inferred AU-rich element is indicated as a green box. The *x*-axis reports time in hours after treatment and the *y*-axis reports expression normalized to the maximum expression in GM10860. Error bars represent standard deviations (*n* = 2).

We next aimed to shed light on the biological context in which differences in regulatory responsiveness could manifest between long and short transcript forms. For this purpose, we focused on natural variation in alternative polyadenylation at the immune regulator IRF5. In response to the bacterial cell wall component lipopolysaccharide (LPS), immune genes undergo an immediate spike in expression, followed by dampening to a more modest steady-state level mediated by the ARE-binding protein TTP [56]–[58]. Motivated by our discovery of a repressive ARE in the differential region of IRF5 (Figure 6A), we hypothesized that the genetically determined production of long and short 3′ mRNA forms of this gene would be associated with different patterns of regulatory behavior after induction. To test this, we treated B-lymphoblastoid cell lines from genetically distinct individuals with LPS and, in each culture, measured the recovery of expression levels of IRF5 transcript forms over time. The results, shown in Figure 7, revealed, after an initial overshoot in expression, a difference of up to 2-fold in the time to reach steady-state expression between long and short mRNA forms, with the long form downregulated to steady-state more quickly after induction as predicted, given the presence of the repressive ARE in the latter transcript. Variation in IRF5 expression recovery across B-lymphoblastoid lines was associated with genotype at the proximal polyadenylation signal in the IRF5 3′ UTR: haplotypes encoding the long form of IRF5 conferred rapid repression after induction (Figure 7B) relative to haplotypes encoding the short form (Figure 7A). These findings suggest that genetic variation in 3′-end processing dictates differences across individuals in the regulatory dynamics of IRF5, further underscoring the power of our approach to identify biologically relevant regulatory effects of 3′-end processing.

### 3′ *cis*-regulatory elements and genetic variation in gene expression

**Figure 8:**
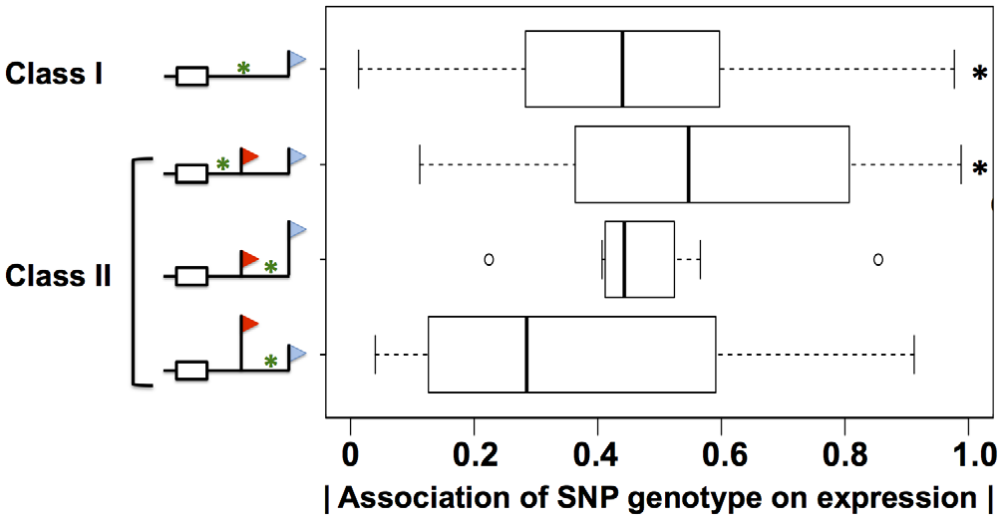
Sequence variation in 3′ motifs as a predictor of expression change across human individuals. Shown are analyses of DNA sequence variants in regions meeting bioinformatic criteria for A/U rich elements, G/U-rich elements, binding sites for the Pumilio family of proteins, and Alu transposable elements, in genes with expression heritability >0.5 (Materials and Methods). Each row represents results from analysis of the set of variants in a particular region of 3′ untranslated regions of genes subject to alternative polyadenylation (symbols and class identifiers as in Figure 1B), denoted by a green asterisk in the schematic at left. In a given class II gene, variants were classified according to their position relative to the proximal and distal 3′ end locations; variants between the two 3′ ends were further classified by the relative abundances of the short and long 3′ transcript forms on average across cell lines, indicated by the heights of the flags. For each variant, analysis comprised a linear regression across genetically distinct individuals between inheritance at the locus and expression of the gene in which it lay; for each row, the *x*-axis reports a distribution of the absolute values of Pearson correlation coefficients from such linear regressions, as in Figure 3A. Black asterisks represent comparisons against the set of all variants in 3′ untranslated regions (Figure 3A) significant at *p*<0.02 by a one-sided Wilcoxon test.

Our molecular confirmation of *cis*-regulatory elements in 3′ UTRs inferred from sequence search methods (Figure 6) suggested that such inference could provide a mechanistic understanding of gene expression on a genomic scale. In particular, we expected that sequence variants between individuals in 3′ regulatory elements would be significant predictors of variation in steady-state expression of the genes in which they lay. To test this notion, we first tabulated all single-nucleotide polymorphisms across the cell lines of our data set which overlapped with 3′ regulatory motifs and Alu elements in 3′ UTRs. We next classified these motifs according to the impact of alternative polyadenylation on their positions in 3′ UTRs, and we used association tests to assess the strength of each motif variant in each class as a predictor of steady-state expression of its respective gene. The results (Figure 8) revealed association with expression across human individuals, for variants in AREs, G/U-rich elements, Pumilio sites, and Alu elements. The relationship with expression was striking and significant for variants in regions of 3′ UTRs constitutively incorporated into mature messages (Figure 8). Polymorphic motifs incorporated into low-abundance 3′ length forms showed no evidence of association with expression changes in their respective genes, consistent with the minor contribution of these forms; by the same token, polymorphic motifs in regions incorporated into the predominant 3′ length forms of mRNAs were more strongly associated with expression of their respective genes, though not significantly so (Figure 8). As expected [6], the relationship between sequence variants and gene expression did not manifest for miRNA sites (data not shown). These findings highlight the relevance of 3′ regulatory motifs as predictors of expression variation across human individuals, when integrated with knowledge of transcript length forms from our sequencing strategy.

## Discussion

Alternative polyadenylation is prevalent in the human transcriptome, and in landmark cases, variation across individuals in the use of 3′ length forms of RNAs has been shown to underlie human disease [15]–[20]. However, for most human genes, the regulatory importance of changes in transcript ends between individuals is incompletely understood, owing to the challenges of measuring 3′-end usage and identifying functional regulatory elements in 3′ UTRs. We have developed a spatially precise, quantitative, high-throughput sequencing approach for 3′ ends, complementing now-classic studies of expressed sequence tags [30], [36], [48], [51], [59]–[61]. We have used the resulting transcript end positions and abundances to pioneer an analysis approach which integrates bioinformatic predictions of 3′ regulatory motifs, genomic analysis, and molecular genetics. With this strategy, we have established a regulatory map of transcript ends and functional elements in the 3′ UTRs of lymphoblastoid cells, and we have abstracted genomic principles of alternative polyadenylation and natural genetic variation in this cell type.

Our mapping of 3′-end length forms and sequence motifs in 3′ UTRs revealed an intuitive logic in which the choice between short and long UTR forms governs the incorporation of regulatory elements into mature messages [36], [61]. We note that the 3′ length forms we report in B-lymphoblastoid cells represent a subset of the total complement of 3′ UTR lengths used across tissues. As such, we hypothesize that surveys of tissue types will ultimately reveal transcript forms of many genes, used in particular contexts, that incorporate 3′ regulatory information to different extents. The ability to tune the responsiveness to *trans*-acting input itself distinguishes alternative polyadenylation from other transcriptional and post-transcriptional regulatory mechanisms, providing a compelling model for the particular advantage of 3′-end processing as a regulatory strategy and a rationale for its prevalence in mammalian genomes.

In comparisons across cell lines from genetically distinct individuals, we analyzed the regulatory importance of genetic changes at 3′ transcript ends. We uncovered a key role for polymorphisms in polyadenylation signals as a driver of changes in gene expression, and we detailed the molecular mechanisms at play. The polyadenylation variants we study here dictate the production of transcript forms with different determinants of 3′-end processing, different half-lives, and different recognition sites for *trans*-acting regulators. These findings establish a connection between observational studies of transcript 3′ length forms across human populations [21], [22] and regulatory effects of this variation. In the case of IRF5, we discovered that a naturally occurring genetic change in usage of 3′ RNA forms can serve to tune the kinetics of recovery of expression after induction by lipopolysaccharide. Thus, against the backdrop of prior studies of this lupus susceptibility gene [15], we have uncovered an additional dimension by which variation in RNA 3′-end processing affects regulatory behavior. Given these case-study results as a validation of our genome-scale analyses, we speculate that many genetic variants with biologically relevant effects mediated by RNA 3′-end processing remain to be discovered in the human population.

We have also shown that polymorphisms in 3′ motifs that govern transcript fate can serve as predictors of steady-state levels of the genes in which they lie. In light of the ultimate goal of predicting regulatory and phenotypic effects from human genome sequence, our results indicate that analysis strategies using sequence determinants of transcription initiation and splicing alone are likely to provide an incomplete model of expression variation. Importantly, however, our work makes clear that binding sites for regulators of mRNA localization, half-life, and translation at 3′ ends are themselves only part of the regulatory landscape. Rather, a complete understanding of the genetics of gene expression will integrate the usage of RNA 3′-end processing signals with the effects of 3′ sequence elements that control transcript fate. We anticipate that abundances and positions of transcript ends observed in 3′-end RNA-seq will prove to be a key component in the systems-level modeling of regulatory networks and their variation.

In summary, while the genetic study of RNA 3′-end processing is in its infancy, our work and that of others [15]–[22] establishes that variation at 3′ ends can be a critical determinant of regulatory behaviors. However, for the vast majority of human genes, the importance of 3′ regulatory change remains unknown. Our single-gene experiments detail the expression effects of 3′ UTR variation in the disease-associated genes IRF5 [15] and DIP2B [55]; the potential for regulatory variants as drivers of human disease will serve as continued motivation for genomic and genetic analyses of expression change.

## Materials and Methods

### Cell culture and RNA extraction

Two biological replicates of each of the human lymphoblastoid cell lines GM10860, GM17106, GM17189, GM17207, GM17220, and GM17253 (Coriell Institute) were cultured in RPMI 1640 medium supplemented with 2 mM L-glutamate and 15% fetal bovine serum. Cells were incubated at 37°C under 5% carbon dioxide. Total RNA was extracted from ∼10^7^ cells using TRIzol reagent (Invitrogen), and genomic DNA was removed from RNA using Turbo DNase (Ambion).

### 3′-end RNA–seq library preparation

Polyadenylated RNA was selected from 10 µg of total RNA using the Dynabeads mRNA purification kit (Invitrogen), and was fragmented for 3 minutes at 70°C using 10× Fragmentation Reagent (Ambion). After ethanol precipitation, polyadenylated RNA fragments were selected using the Dynabeads mRNA purification kit and reverse transcribed using SuperScript II (Invitrogen) and anchored oligo-dT (Invitrogen). Double-stranded cDNA was generated using RNase H (Invitrogen) and DNA Pol I (Invitrogen), end-repaired using T4 DNA Polymerase (New England Biolabs), Klenow DNA Polymerase (New England Biolabs), and T4 PNK (New England Biolabs), and then adenylated using Klenow 3′ to 5′ exo minus (New England Biolabs). Illumina paired-end adapters were ligated to the adenylated cDNA using T4 DNA Ligase (Enzymatics). Ligated cDNA was purified on a 2% agarose gel and then amplified by performing 12 cycles of PCR using Phusion HF Polymerase (New England Biolabs). Libraries were sequenced using 40 bp paired-end modules on an Illumina 2G Genome Analyzer.

### Read filtering and mapping

Consecutive T's from the beginning of all reads were trimmed and classified before mapping: for a given read, if there were more than 20 consecutive T's or if neither mate of a read pair had a stretch of T's, the read was not included in further analysis. For mapping, the set of single nucleotide polymorphisms (SNPs) segregating in the CEU population was downloaded from HapMap phase II+III, release 27 (ftp://ftp.ncbi.nlm.nih.gov/hapmap/genotypes), and used to modify the human reference genome (hg18; [62]) by incorporating the appropriate ambiguous bases at each SNP position. All reads were then mapped to this modified genome and to the associated splicing junctions in the Known Genes database of the UCSC genome browser [63] using MOSAIK (http://bioinformatics.bc.edu/marthlab/Software_Release). For a given length of trimmed T's, *n_T_*, a splicing junction reference was created by concatenating 37 - *n_T_* bp from each exon adjacent to the splicing junction to ensure that the reads mapped across the splicing junction. If the reads mapped to both the genome and a splicing junction, the mapping with smaller number of mismatches was used. Only uniquely mapped reads with two or fewer mismatches in each mate were retained. Trimmed T's were then compared to the genome sequence; reads with >2 mismatches to the genome in this poly-T tract were retained for analysis. We inferred that a given read was transcribed from the minus strand of the genome if, when it was mapped to the reference genome, the position of its poly-T tract had a lower coordinate position than the mapped position of the other end of the read; we inferred that a read was transcribed from the plus strand of the genome if the mapped position of its poly-T tract had a higher coordinate position than the position of the other end. Mapped reads yielded an average coverage of 22.6% of UCSC annotated 3′ UTRs with a depth of 97.8 reads/bp for the covered bases for each sample. The last 100 bp of annotated 3′ UTRs were even more highly represented in libraries, with an average coverage of 43.4% and an average depth of 211.1 reads/bp.

### Defining tag clusters

Mapped reads from all samples were pooled, sorted according to the polyA positions, defined as the coordinate of the base adjacent to the polyA tail, and then grouped into tag clusters as follows. For each strand of each chromosome, the 5′ boundary of a tag cluster was set as the polyA position of the first read, and then reads were sequentially added to this unit until the polyA position of the next read was more than 15 bp away. The latter position then became the 5′ boundary of the next tag cluster.

Most tag clusters spanned less than 24 bp, but if the polyA positions in a cluster spanned more than 40 bp, we applied a peak-finding algorithm as follows. For each genome coordinate in the region corresponding to the tag cluster, we defined the read count as the number of reads whose polyA position overlapped the coordinate. From these we first identified the genome coordinate (*posM*) with the greatest read count (*MaxHeight*). We then delineated a window 40 bp upstream and 40 bp downstream of this coordinate. Within this window, we retained all coordinates with read counts greater than 10% of *MaxHeight*. Of these, we identified the most 5′ and 3′ polyA positions (*posL* and *posR*). All reads in the tag cluster were then divided into three new candidate tag clusters: reads with positions 5′ to *posL*, reads with positions including and between *posL* and *posR*, and reads with positions 3′ to *posR*. If the distance between *posL* and *posR* was longer than 40 bp, the middle candidate tag cluster was eliminated from further analysis. If the read counts of all coordinates in a candidate tag cluster were below 10% of *MaxHeight*, the candidate unit was eliminated. If a candidate tag cluster contained coordinates with read counts larger than 10% of *MaxHeight*, we identified the coordinate with the largest read count within this candidate tag cluster and repeated the peak-finding algorithm. For each tag cluster retained for analysis, we defined the polyA position of the unit as the median of the polyA positions of all reads encompassed by the unit.

After establishing that the stretch of A's in each read represented a mismatch to the genome sequence (see above), we filtered out reads with a potential origin from internal priming from A-rich regions of the genome by removing any tag cluster whose defined polyA position was followed by 10 or more A's in the genome sequence within 20 bp. We also filtered out reads with a potential origin as PCR clones or false mapping as follows. We expected that for a given set of paired-end reads falling into a tag cluster, the characteristics of read ends originating from biological 3′-end processing should be distinct from the characteristics of read ends originating from fragmentation, reverse transcription, and ligation during RNA-seq library preparation. In particular, we reasoned that the polyA positions of a set of reads in a tag cluster with origin as a biologically relevant transcript would be less heterogeneous than the positions of the other mates of the reads. If the opposite were true, we considered the tag cluster to be a likely product of false mapping or PCR duplication. As such, we retained a tag cluster for analysis only if the precision of polyA positions across the reads of the unit was greater than the precision of the positions of the other mates. The precision was calculated by
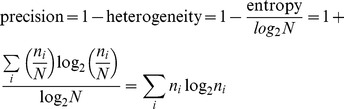
 where *n_i_* is the number of reads at the *i*th position and *N* is the total number of reads in the tag cluster. We also filtered out any tag cluster whose total read count across all samples amounted to fewer than 50 reads. For tag clusters with read counts between 50 and 100, we calculated the Pearson correlation coefficient *R* between each pair of the two biological replicates across the six cell line samples, and eliminated the tag cluster from further analysis if the absolute value of *R* was less than 0.5.

### Consensus sequences

For use in searches for regulatory motifs, we harnessed all 3′-end RNA-seq reads in tag clusters from all samples to define a consensus base at each position in 3′ UTRs as follows. At every genomic coordinate covered by five or more 3′-end RNA-seq reads, the consensus nucleotide was chosen as that with highest frequency across the sample. If the second most abundant base was more than 20% in abundance, it was incorporated into the consensus using an ambiguous base notation (M = A or C, R = A or G, W = A or T, S = C or G, Y = C or T, K = G or T).

### Identification of polyadenylation signals and auxiliary elements

For every tag cluster, the consensus sequence of the region 40 bp upstream from the polyA position was searched for a polyadenylation signal using the known hexamer motifs sorted by their abundance in the human genome from [30]. Polyadenylation signals with higher abundance were given higher priority when there was more than one instance in the 40 bp upstream window. In the absence of a match to these motifs, the 40 bp upstream window was searched for an A-rich stretch as follows. We identified any 9 bp regions containing at least 6 A's and considered each such region a candidate polyadenylation signal; if no such candidate signal were present, we considered the tag cluster not to have an identifiable polyadenylation signal. If more than one candidate signal was present, we retained the one with the most A's. For each boundary of this region, if the boundary nucleotide was an A, the region was extended to include all consecutive A's. If the first base was not an A, it was trimmed until the first base was an A.

To find upstream U-rich elements (USE), for a given tag cluster, we identified a candidate USE as the 9-bp window with the highest number of T's in the consensus sequence, within 30 bp upstream of the polyadenylation signal. If there were fewer than six T's or if the candidate did not have three consecutive T's, we considered the tag cluster not to have an identifiable USE. For each boundary of a given candidate window, if the boundary nucleotide was a T, the window wasextended if there was a T in the adjacent 2 bp and the proportion of T's in the window was above 65%; if the boundary base was not a T, we trimmed the candidate window until a T was reached.

To find downstream U/G-rich elements (DSE), for a given tag cluster, we identified a candidate DSE as the 9-bp window with the highest number of T's in the consensus sequence, within 40 bp downstream of the poly-A position. If there were fewer than five T's or if the window did not contain at least one of the strings TTT, TGTG, GTGT, GTCT, CTGT, TCTG, or TGTC, we considered the tag cluster not to have an identifiable DSE. For each boundary of a given candidate window, if the boundary nucleotide was a T, the window was extended if there was a T in the adjacent 2 bp and the proportion of T's in the window was above 50%; if the boundary base was not a T, we trimmed the candidate window until a T was reached.

### Comparison of tag cluster positions with UCSC annotations and polyA_db2

Human gene annotations were downloaded from the Known Genes database of the UCSC Genome Browser [63]. Among the UCSC transcript annotations that overlapped the start and end positions of a tag cluster, the annotation with the minimum distance between the annotated 3′-end position and the polyA position of the tag cluster was chosen. If there were multiple annotations with the same 3′-end positions, we chose the annotation with greatest degree of overlap between the tag cluster consensus sequence and the annotated exons.

The genomic coordinates of human expressed sequence tags (ESTs) with polyA tails were downloaded from the polyA_db2 database [64]. The genomic coordinates were converted from hg17 to hg18 using the liftOver tool from the UCSC Genome Browser [65]. If the coordinate of polyA_db2 EST was between the start and end position of a tag cluster, we considered the length form corresponding to the unit to be supported by the EST.

### Classification of alternative polyadenylation

All tag clusters with the same UCSC gene annotation were categorized as associated with the gene, and only the two tag clusters with the highest expression per gene were used for classification of alternative polyadenylation. We classified each gene as follows: class I if there was only one tag cluster overlapping with the annotated 3′ UTR of the gene; class II if both tag clusters overlapped with the annotated 3′ UTR of the gene and they were associated with the same UCSC transcript ID; class III if one of the tag clusters overlapped witha coding exon or intron, or if both tag clusters overlapped with 3′ UTRs with different UCSC transcript IDs.

### Heritability of polyA position and expression

We calculated the broad-sense heritability in polyA positions using biological replicates of 3′-end RNA-seq across the six human cell lines as follows. For a given gene, we considered the polyA position of the most abundant tag cluster in each replicate of each sample as a quantitative trait, and calculated the heritability *H^2^* of this trait from intraclass correlations [66] given by the following equations.
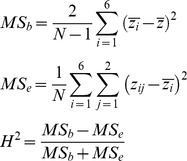
 Here, indices *i* and *j* refer to sample and replicate, respectively; 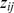 is the polyA position for sample *i*, replicate *j*; 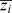 is the average between two replicates for sample *i*; 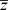 is the average of all samples and replicates; *MS_e_* is the error mean square (within-individual) and *MS_b_* is the between-individual mean square. Heritability of total expression levels for each gene was calculated analogously, using as a quantitative trait the sum of read counts across the gene normalized by the sum of all reads.

### Regulatory element motif searches

To identify microRNA binding sites in 3′ UTRs, the Perl script from TargetScan Release 5.2 [67] was used to predict miRNA binding sites in the 3′ UTR sequences of all expressed genes. miRNA sequences and families were downloaded from the TargetScan database. Only predicted binding sites with context scores less than −0.4 were used for analysis. To identify AU-rich elements, the class II motif WWWT(ATTTA)TTTW was searched in the 3′ UTR sequences of all expressed genes allowing up to one mismatch outside the central pentamer, ATTTA. Overlapping motifs were combined. To identify GU-rich destabilizing elements, the motif TGTTTGTTTGT was searched in 3′ UTR sequences allowing up to one mismatch. To identify Pumilio binding elements, the motif TGTANATA was searched in 3′ UTR sequences. Alu transposable element motifs were taken from RepeatMasker (www.repeatmasker.org) downloaded from the UCSC Genome Browser. We note that each search strategy used human sequence data alone rather than inter- or intra-species conservation to identify motifs.

### Conservation of regulatory element motifs in the human population

SNPs within the human population were downloaded from the 1000 Genomes Project database [68]. Genomic coordinates were converted from hg19 to hg18 using the liftOver tool from the UCSC Genome Browser [65]. For each regulatory element motif, the SNP rate was calculated as the total number of SNPs within all motif matches divided by the sum of the lengths of all matches.

For the background model of sequence variation in 3′ UTRs used in Table S4, we first tabulated all instances of A, C, T, and G across all positions of 3′ UTR sequences in the hg18 human reference genome, where the boundaries of 3′-ends were taken from our compendium of 3′-end RNA-seq data for genes expressed in our samples. We refer to these frequencies as *P(A)*, *P(C), P(T)*, and *P(G)*, respectively. We then tabulated the SNP rate separately for each of these four sets of base positions from the 1000 Genomes data set, which we refer to as *P*(*SNP*, *A*), *P*(*SNP*, *C*), *P*(*SNP*, *T*), and *P*(*SNP*, *G*), respectively. We used these values to calculate the expected density of polymorphisms for the stretch of genome corresponding to a given 3′ motif match as:
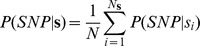
 where 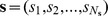 is the sequence of bases of length *N* **_s_** corresponding to the motif match in the human reference genome and *P*(*SNP*|*s_i_*) is the probability of a single-nucleotide polymorphism for the nucleotide *s_i_* at position *i*, calculated as:
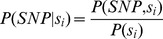
 For each regulatory motif, *P*(*SNP*|**s**) was calculated for every instance of a motif match across all 3′ UTRs in the reference genomefor genes expressed in our samples, and the lower and upper bounds listed in Table S4 were taken as the minimum and maximum values of this distribution, respectively. For miRNA binding sites, only the 7-mer seed sequence was used.

### Gene Ontology analysis of genes with alternative polyadenylation

The DEFOG web-based tool (http://www.mooneygroup.org/defog) was used for Gene Ontology term enrichment analysis in Table S5 as follows. For each type of regulatory element analyzed in Figure 2C, we tabulated a list of class II genes with motifs in the shared region only, and combined these lists across elements. For each Gene Ontology term, we then evaluated the significance of the representation of genes annotated in the term in this list, relative to a background set of class II genes harboring at least one motif, using DEFOG with default parameters. Separately, we tabulated an analogous combined list of class II genes with motifs in the differential region only and repeated the DEFOG analysis.

### Association between 3′ UTR sequence variants and gene expression

To find polymorphisms in 3′ UTRs across the six individuals of our sample, we generated mRNA-seq libraries [69] from one biological replicate of each cell line sample, and sequenced using 36 bp paired-end modules on an Illumina 2G Genome Analyzer, resulting in 21 to 24 million reads per sample. The sequenced reads were mapped to the human genome (hg18) using MOSAIK (http://bioinformatics.bc.edu/marthlab/Software_Release). Mapped reads (10 to 14 million per sample) yielded an average coverage of 48% of UCSC annotated exons with an average depth of 27 reads/base for the covered bases. Mapped reads were used to call SNPs using GigaBayes (http://bioinformatics.bc.edu/marthlab/Software_Release) with options –ploidy diploid –O 3 –indel –CAL 10. Only SNPs with a quality score higher than 0.99 were retained for analysis. This set of SNPs was used in association tests with total gene expression levels as follows. Given tag cluster definitions for each gene from analysis of 3′-end RNA-seq libraries (see above), we calculated a normalized expression level for each tag cluster in each sample as the ratio between the number of 3′-end RNA-seq reads mapping within the tag cluster boundaries in the sample and the total number of mapped 3′-end RNA-seq reads in the sample. We then defined the expression level of a given gene in a given sample as the sum of all normalized expression levels across all tag clusters in that gene. To get a final estimate of gene expression level for use in association tests in Figure 3 and Figure 8, we summed expression values across the two replicate samples from each cell line.

For each SNP in the 3′ UTR of a gene, we identified the major allele across all cell lines and scored each diploid genotype in terms of the number of major alleles (values ranging from 0 to 2). Given the complete matrix of gene expression levels and genotypes across all six cell lines, we calculated the Pearson correlation coefficient *R* between allele counts and the started logarithm of the gene expression level. For Figure 3A and Figure 8, we used the absolute value of *R* as the association statistic, and for Figure 3B, we used the signed value of *R*. In cases of alternative polyadenylation, SNPs in polyadenylation signals upstream of a maximum of two major length forms were considered for association tests. To analyze the effect of genetic variation in polyadenylation signal strength on expression in Figure 3B, each allele of each polyadenylation signal was assigned a score: 1 if the signal was AATAAA or ATTAAA, 0.5 if the signal was a match to the “variant” polyadenylation motifs in [30], and 0 otherwise. The polyadenylation signal strength difference was calculated by subtracting the strength of the minor allele from the major allele. In Figure 3A, we analyzed SNPs in 3′ UTRs for 4214 genes and SNPs in polyadenylation signals for 33 genes. In Figure 8, we analyzed regulatory element SNPs in 62, 16, 7, and 20 genes respectively in 3′ UTRs of class I genes, shared regions of 3′ UTRs of class II genes, differential regions of 3′ UTRs of class II genes whose long forms were more abundant, and differential regions of 3′ UTRs of class II genes whose short forms were more abundant.

### Cloning of 3′ UTR reporter vectors

A psiCheck-2 vector (Promega) was modified to generate a 3′ UTR reporter vector, pOKY001 (Figure S5 and Table S8), in which we removed the SV40 late polyA signal from the firefly luciferase gene and replaced it with a tag for ligation independent cloning (LIC) [70] of 3′ UTR sequences of interest. Genomic DNA was isolated from lymphoblastoid cell lines using the MasterPure DNA Purification Kit (Epicentre), and 3′ UTR sequences were amplified from genomic DNA using Phusion HF Polymerase (New England Biolabs) with PCR primers with LIC tags. PCR products were cloned into pOKY001 using LIC, and plasmids were purified using Plasmid Midi Kit (Qiagen). Site-directed mutagenesis of plasmids was performed using QuikChange XL Site-Directed Mutagenesis Kit (Agilent) according to the manufacturer's instructions. All primers used for PCR and mutagenesis are listed in Table S7. Cloned sequences of all reporter vectors were checked by capillary sequencing. The list of reporter vectors is provided in Table S8.

### Transfection of 3′ UTR reporter vectors

HEK293T cells were cultured in Dulbecco's modified Eagle's medium with 10% fetal bovine serum and 1% non-essential amino acids in six-well culture plates. For each of two independent transfections for each construct, at 90% confluency, 1 µg of plasmid DNA was transfected using Lipofectamine 2000 (Invitrogen) according to the manufacturer's instructions. After incubating at 37°C under 5% carbon dioxide for 24 h, the cells were washed with Dulbecco's Phosphate-Buffered Saline (DPBS), detached from plates by adding 300 µL of trypsin-EDTA and incubated at 37 degrees for 5 minutes. After adding 900 µL of media, the detached cells were split into three 2 mL tubes, and pelleted by spinning at 1000× g for 3 minutes. The cell pellets were washed with DPBS and then flash-frozen in liquid nitrogen and stored at −80°C.

### Luciferase measurements

Given one cell pellet from each of two reporter transfections (see above), cells from each pellet were lysed and used for two technical replicates of a dual luciferase assay using the Dual Luciferase Reporter Assay System (Promega). Luminescence from the activities of firefly and Renilla luciferases were measured sequentially in a Turner BioSystems Veritas Luminometer. The ratio of luminescence measurements between Firefly and Renilla luciferases was used for all analyses.

### Quantitative PCR

To measure expression of short and long forms for a given gene with alternative polyadenylation, we considered quantitative PCR assays that would interrogate regions just upstream of the inferred cleavage sites of the short and long forms (which we refer to below as SU and LU, respectively). We reasoned that expression measurements of SU would reflect abundance of both the short and long transcript forms, whereas LU would reflect abundance of the long form only. For a given transcript in a given experiment, the absolute mRNA level (calculated from quantitative PCR reactions on a biological sample and standards, as described below) from the primer set amplifying LU was used as the expression level for the long form; the expression level of the short form was calculated by subtracting the absolute mRNA level of LU from the absolute mRNA level of SU for all genes except IRF5. Primer sequences for IRF5 were taken from [15], where the primer set for SU hybridized to the polyA tail and only amplified the short form. Thus, for IRF5, the mRNA count from SU was used as the expression level for the short form. All other primers were designed using Primer3 Plus [71], checked for potential hairpins and primer dimers using BeaconDesigner Web Edition (PREMIER Biosoft International). Primers were synthesized by Elim Biopharmaceuticals. For each transcript in each experiment, measurements were normalized by absolute mRNA counts of Renilla luciferase or GAPDH genes. All primer sequences are in Table S7.

Expression measurements on cells transfected with 3′ UTR reporters were performed as follows. Given one cell pellet from each of two transfections (see above), total RNA was isolated from each pellet separately using TRIzol (Invitrogen). For each sample, genomic DNA was removed from 10 µg of total RNA using TurboDNase (Ambion). Single-stranded cDNA was synthesized from 2 µg of DNased RNA using oligo(dT) (Invitrogen) and SuperScript III reverse transcriptase (Invitrogen), and then RNA was removed using RNase H (Invitrogen). For each of two technical replicates for each primer set and sample, the cDNA was amplified using the DyNamo HS SYBR Green QPCR Kit (Thermo Scientific) for 40 cycles on a Strategene MX3000P qPCR instrument. We calculated absolute numbers of mRNA molecules amplified from each primer set in each experiment by comparing the number of amplification cycles taken to reach a given threshold against a standard curve constructed using samples of a synthetic template (gBlocks Gene Fragments, Integrated DNA Technologies) of known absolute numbers of molecules, in serial dilutions extending from 10^7^ to 10^1^ molecules.

### 3′ processing signal reporter design

Reporters incorporating 3′ processing signals were constructed as follows. For each length form of each gene, we aimed to clone a region centered on the cleavage site as inferred from the transcript end position observed in 3′-end RNA-seq, bounded by the 40 bp upstream and 40 bp downstream in the genomic sequences and flanked by LIC tags (region sequences for EIF2A, IRF5, DIP2B, and NAB1 were taken from the haplotype giving rise to production of both long and short forms for cell lines GM17220, GM10860, GM10860, and GM17220 respectively). This 115-bp construct was produced by PCR stitching two opposite-strand DNA oligos of 60 bp and 78 bp (Integrated DNA Technologies) with 23 bp overlap using Phusion HF Polymerase (New England Biolabs) for 3 cycles. PCR products were cloned into pOKY001 and purified as described above. Transfection and dual luciferase assay were performed as above.

### Transcript half-life measurements

For each transcript half-life measurement, cells transfected with a luciferase reporter harboring the complete 3′ UTR from the haplotype giving rise to production of both long and short forms of a given gene were incubated at 37°C under 5% carbon dioxide for ∼24 h. A stock solution of actinomycin D dissolved in DMSO was added to achieve a final actinomycin D concentration in tissue culture media of 10 µg/mL. After incubation times as indicated in Figure 5 and Figure S4, cells were harvested for quantitative PCR measurements as above or Northern blotting as described below.

### Dose-response for trans-acting factors

To generate expression vectors for ARE-binding proteins, the tristetraprolin (gene name ZFP36) coding sequence was amplified from the vector pGFP-TTP and the AUF1 p37 coding sequence from pCDEF-His-AUF1-p37 (both kind gifts from B. Glaunsinger). Each gene was cloned into the pcDNA3 mammalian expression vector (Invitrogen). Syn-has-miR-101, a miRNA mimic of has-miR-101, was purchased from Qiagen (MSY0000099). For dose-response assays, expression vectors or miRNA mimic were mixed with the corresponding luciferase reporter vectors (Figure 6) and then transfected as above.

### IRF5 induction with lipopolysaccharide

Lipopolysaccharide from *E. coli K12* (Invivogen) was added to each of the human B-lymphoblastoid cell lines GM10860, GM17106, and, GM17207 to a final concentration of 5 µg/mL in 6-well tissue culture plates, and incubated at 37°C under 5% carbon dioxide. The cells were harvested after 0, 1, 2, 4, 6, 8, 12, and 16 hours by centrifuging at 1000× g for 3 minutes, washing with DPBS, and then flash-freezing in liquid nitrogen. RNA was isolated using Trizol (Invitrogen).

### Northern blotting

The DIG Northern Starter Kit (Roche) was used for Northern blotting. A 400 bp region of firefly luciferase gene was amplified from luciferase reporter vector pOKY001, and cloned into pcDNA3 vector (Invitrogen). A digoxigenin (DIG) labeled RNA probe for firefly luciferase was created by *in vitro* transcription with SP6 RNA polymerase after linearizing the plasmid with BamHI. Total RNA isolated using Trizol (Invitrogen) was cleaned using RNeasy columns (Qiagen) with on-column DNase-digestion. Between 1 and 10 µg of total RNA was loaded and run on 1% denaturing agarose gels. The gels were stained with SYBR Gold (Invitrogen) and then the separated RNA was blotted onto nylon membranes (Roche) and UV cross-linked. Both the gels and the membranes were imaged on Blue Light Transilluminator (Invitrogen) to check the transfer of SYBR-stained RNA. 28S ribosomal RNA bands on the membranes were used as the loading control in Figure S4. Each membrane was subjected to prehybridization, probe hybridization, low and high stringency washing, and detection procedures recommended by the manufacturer (Roche). Anti-digoxigenin-AP (Roche) and CDP-Star (Roche) were used for chemiluminescent detection of the DIG-labeled RNA probe. To maximize length resolution for EIF2A, total RNA was incubated at 37°C for 30 min with RNaseH (Invitrogen) and an oligo antisense to a 20 bp region upstream of the binding site for the Northern probe in the firefly gene. For DIP2B, the denaturing agarose gel was treated with 0.05% NaOH and washed with water before blotting onto the nylon membrane.

### Accession numbers

The expression data reported in this paper have been deposited in the Gene Expression Omnibus (GEO) (http://www.ncbi.nlm.nih.gov/geo) database (series accession number GSE33154).

## Supporting Information

Figure S1 Reproducibility of 3′-end RNA-seq. Each panel reports a comparison between two 3′-end RNA-seq data sets. Each data point reports abundance for a set of 3′-end RNA-seq reads defined as a transcript end (a tag cluster; Materials and Methods); axes report the sum of such reads normalized by library size for each of two replicates (Rep 1 and 2, respectively) for each of six B-lymphoblastoid cell lines as indicated. Pearson correlation coefficients are shown at top right. Biological replicate comparisons are indicated in red.
(TIFF)

Figure S2 Quantitative agreement of transcript end positions between 3′-end RNA-seq and annotated coding genes. Shown is analysis of each group of 3′-end RNA-seq reads defined as a transcript end (a tag cluster; Materials and Methods) whose mapped position lay within a 3′ untranslated region of a coding gene in the UC Santa Cruz human genome annotation [63]. The *x*-axis reports the distance between the 3′ boundary of each RNA-seq transcript end form and the 3′ boundary of the annotated untranslated region, and the *y*-axis shows the proportion of all length forms observed in the 3′-end RNA-seq data set with distance from the annotation corresponding to the value on the *x*.
(TIFF)

Figure S3 A-rich stretches as non-canonical polyadenylation signals. (A) At top, cartoons are schematics of alternative polyadenylation with symbols as in Figure 1B. Below, analysis of the inferred polyadenylation signals for each group of 3′-end RNA-seq reads defined as a transcript end (tag cluster; Materials and Methods). Each panel reports categorization of 3′ transcript forms with respect to the presence of polyadenylation sequence motifs upstream of inferred cleavage positions, for the set of genes with the indicated patterns of alternative polyadenylation. Canonical, A(A/U)UAAA; variant, one of 10 variants of the canonical motif, taken from [30]; A-rich, a match to the A-rich non-canonical polyadenylation motif (Materials and Methods); unknown, no match to any polyadenylation motif. “Proximal” and “Distal” represent polyadenylation signals upstream of proximal and distal transcript end forms, respectively. (B) Each panel represents analysis of the set of genes with evidence for alternative polyadenylation (class II or III; see Figure 1B in main text). Each column represents the distribution of 3′-end RNA-seq read counts, across all replicates and samples, in a set of transcript forms defined by the position of the transcript end in the 3′ untranslated region (left) or polyadenylation signal motif upstream of the cleavage site (right). For each distribution, the median is reported as a thick horizontal line, the 25% quantile is shown as a box, and the extremes are shown as thin horizontal bars. Asterisks represent comparisons significant by a two-sided Wilcoxon test at *p*<4×10^−6^. (C) Each row represents luciferase activity in HEK293T cells transfected with one reporter. Reporter schematics are at left; black nucleotides indicate inferred A-rich non-canonical polyadenylation signals in the human reference genome, and red nucleotides indicate mutated sites. Asterisks represent results significant at *p*<0.05 by a two-sided Wilcoxon test comparing the ratio of abundances between the indicated reporters, and error bars represent standard deviations (*n* = 4).
(TIFF)

Figure S4 Variation in 3′ RNA length form usage assayed by Northern blot. Each row reports abundance of 3′ RNA length forms for one gene at which 3′ end usage varied across human B-lymphoblastoid cell lines from genetically distinct individuals (Figure 4). Cartoons at top represent luciferase reporters (hLuc) incorporating the complete 3′ untranslated region of the indicated gene, using a human haplotype producing both short and long 3′ forms (flags) or one bearing a naturally occurring allele that eliminates a canonical polyadenylation signal at one of the two termination sites (red X). For each experiment, a reporter was transfected into HEK293T cells which were then treated with actinomycin D for the indicated length of time (right panels) or untreated (left panels). In each panel, top images report intensities of hybridization of a colorimetric probe complementary to the reporter, and bottom images report intensities of 28S ribosomal RNA by SYBR Gold stain as a loading control. (A) EIF2A, (B) IRF5, (C) DIP2B, (D) NAB1.
(TIFF)

Figure S5 Schematic of cloning and reporter vector pOKY001. LIC, ligation independent cloning.
(TIFF)

Table S1 Mapping and filtering of 3′-end RNA-seq reads. (A) Each value represents the number of reads in the indicated 3′-end RNA-seq library mapping according to the indicated criterion. (B) Each row represents one set of outcomes from filters for stable transcript forms in the analysis pipeline for 3′-end RNA-seq. The first through fourth columns represent filtering steps for tightly grouped reads, PCR cloning or mapping artifacts, mispriming, and abundance, respectively, as described in Materials and Methods. The remaining columns report the number of grouped reads (tag clusters; see Materials and Methods) and the total counts of reads with filter outcomes as indicated, and their overlap with annotated transcript end forms from PolyADB_2 [64]. (C) Each row represents the overlap between tag clusters and reads from 3′-end RNA-seq and one class of genomic feature in the UC Santa Cruz human genome annotation [63]. chrM, features encoded in the mitochondrial genome.
(XLSX)

Table S2 Transcript end positions inferred by 3′-end RNA-seq. Each row reports observations of transcript ends at one human gene. Chrom, chromosome. APA, the classification of alternative polyadenylation as in Figure 1B. Coord, position of the inferred cleavage site for the indicated 3′ length form. TPM, read count normalized by the total number of mapped reads in the library, in tags per million, averaged across all cell lines and replicates in this study. Ratio long/short, ratio of read counts for the long over those for the short form, averaged over all cell lines and all replicates.
(XLSX)

Table S3 Classification of genes according to alternative polyadenylation. Each column represents one pattern of alternative polyadenylation. Classes are defined as in Figure 1B of main text; class III-S indicates genes with only one 3′ length form which did not lie within an annotated 3′ untranslated region according to the UC Santa Cruz human genome annotation [63]. Bold and standard type represent numbers and proportions, respectively, of the indicated features falling into the indicated class.
(XLSX)

Table S4 The density of polymorphisms in 3′ regulatory motifs falls below levels predicted by a 3′ UTR background variation model. Each row represents sequence variation across human individuals from the 1000 Genomes project, in all motif matches meeting bioinformatic criteria for the indicated 3′ element as described in Materials and Methods. Number of SNPs/kb, the observed density of single nucleotide polymorphisms in the indicated motif match regions, as in Figure 2A of the main text. Expected number of SNPs/kb, the expected lower and upper bounds, as indicated, of the density of SNPs in the indicated set of motif matches, predicted based on a background model (Materials and Methods) that incorporates the sequence composition of each motif match in the human reference genome and the estimated likelihood of a SNP at each base.
(XLSX)

Table S5 Genes subject to alternative polyadenylation with regulatory elements in shared or differential regions are enriched for particular gene functions. Each section reports results from analysis of a set of class II genes (see Figure 1B of main text for definitions) with particular characteristics of *cis*-regulatory elements in 3′ untranslated regions (UTRs). The set of genes harboring miRNA sites, AREs, GREs, PUF sites, or Alu elements in shared regions of 3′ UTRs alone, and separately, the set of genes harboring such elements in differential regions alone (see Figure 2B of main text for definitions), was tested for enrichment of Gene Ontology (GO) annotations relative to the complete set of genes harboring elements in any position in the 3′ UTR. *p*-value, results of a Fisher's exact test for over-representation of genes in the indicated GO term in the indicated gene set, corrected for multiple testing by the Benjamini-Hochberg method. Only GO terms with enrichment significant at corrected *p*<0.05 are reported.
(XLSX)

Table S6 Genes with highly heritable variation in usage of 3′ ends across human individuals. Classes are as in Figure 1B of main text. *H* ^2^, broad-sense heritability of 3′-end position calculated as described in Materials and Methods; SNP in PAS, dbSNP identifier of a single-nucleotide polymorphism in a proximal or distal polyadenylation signal as indicated.
(XLSX)

Table S7 Primer sequences used in this work.
(XLSX)

Table S8 Luciferase reporter vectors used in this work.
(XLSX)
