# Supplementary figures and images for: Genetics and Regulatory Impact of Alternative Polyadenylation in Human B-Lymphoblastoid Cells
Source: PLoS Genet. 2012 Aug 16;8(8):e1002882. doi: 10.1371/journal.pgen.1002882 (PMC3420953; doi:10.1371/journal.pgen.1002882)

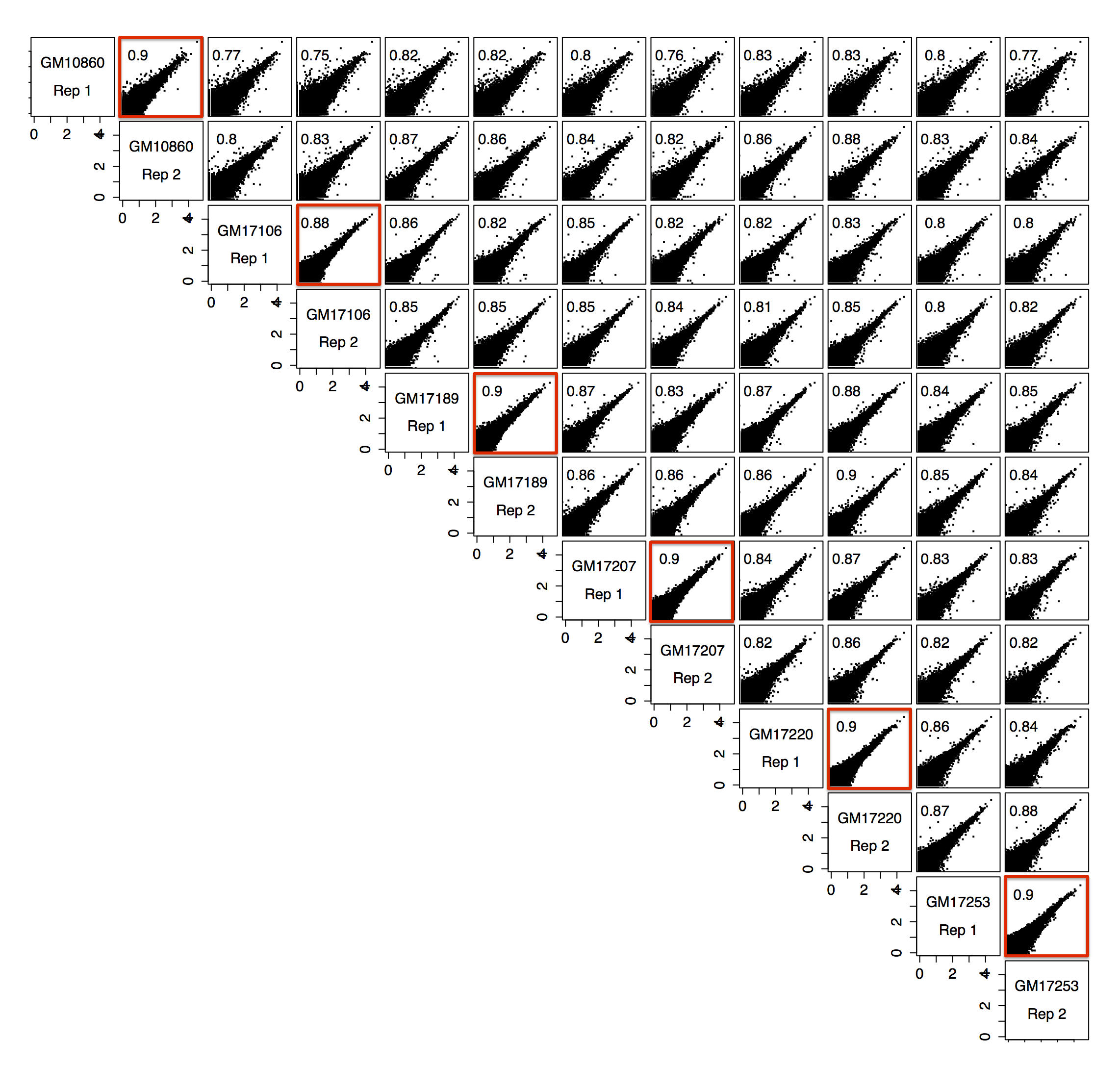

Supplement: Figure S1 — Reproducibility of 3′-end RNA-seq. Each panel reports a comparison between two 3′-end RNA-seq data sets. Each data point reports abundance for a set of 3′-end RNA-seq reads defined as a transcript end (a tag cluster; Materials and Methods); axes report the sum of such reads normalized by library size for each of two replicates (Rep 1 and 2, respectively) for each of six B-lymphoblastoid cell lines as indicated. Pearson correlation coefficients are shown at top right. Biological replicate comparisons are indicated in red. (TIFF) [file pgen.1002882.s001.tiff]

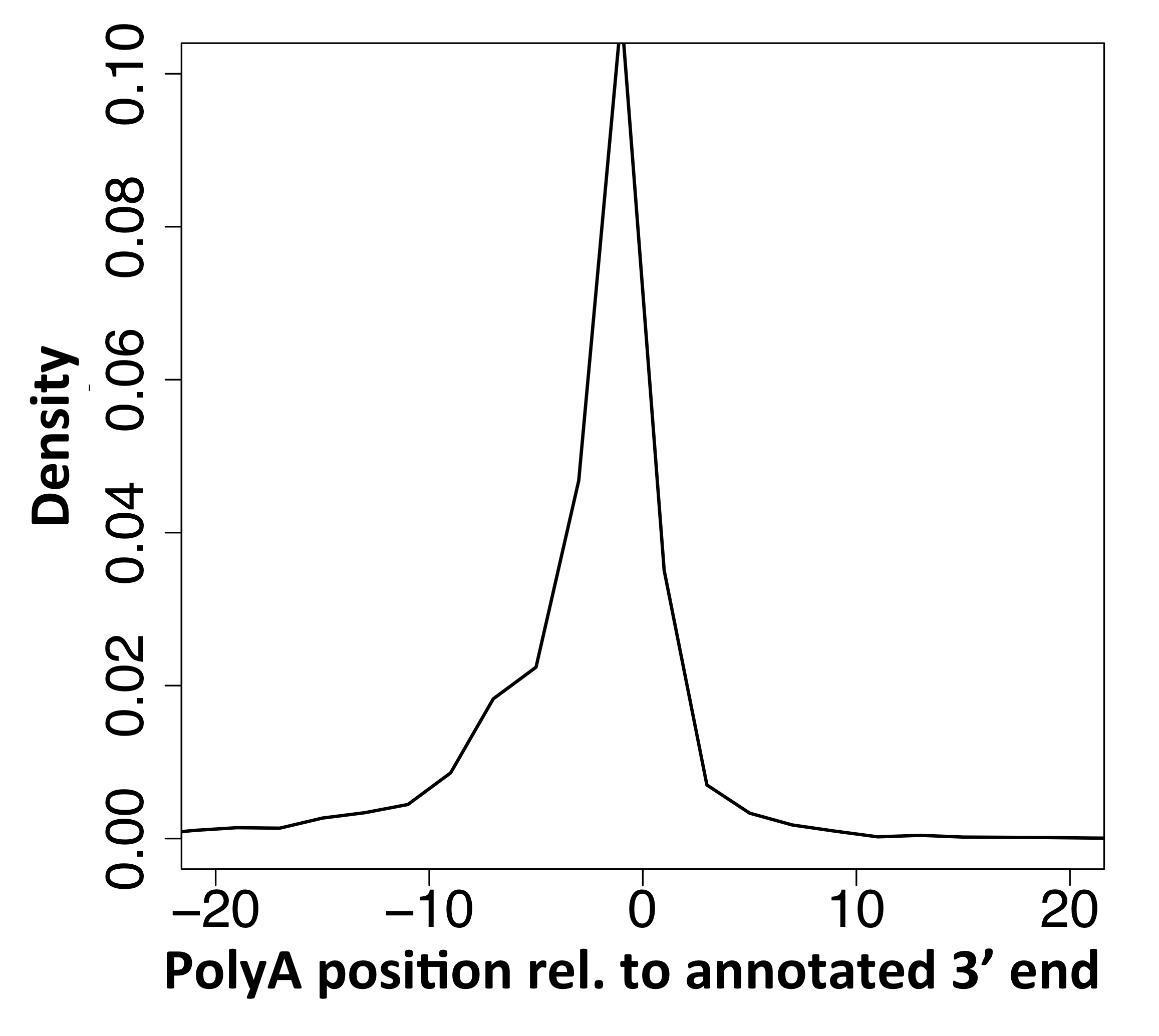

Supplement: Figure S2 — Quantitative agreement of transcript end positions between 3′-end RNA-seq and annotated coding genes. Shown is analysis of each group of 3′-end RNA-seq reads defined as a transcript end (a tag cluster; Materials and Methods) whose mapped position lay within a 3′ untranslated region of a coding gene in the UC Santa Cruz human genome annotation [63]. The x-axis reports the distance between the 3′ boundary of each RNA-seq transcript end form and the 3′ boundary of the annotated untranslated region, and the y-axis shows the proportion of all length forms observed in the 3′-end RNA-seq data set with distance from the annotation corresponding to the value on the x. (TIFF) [file pgen.1002882.s002.tiff]

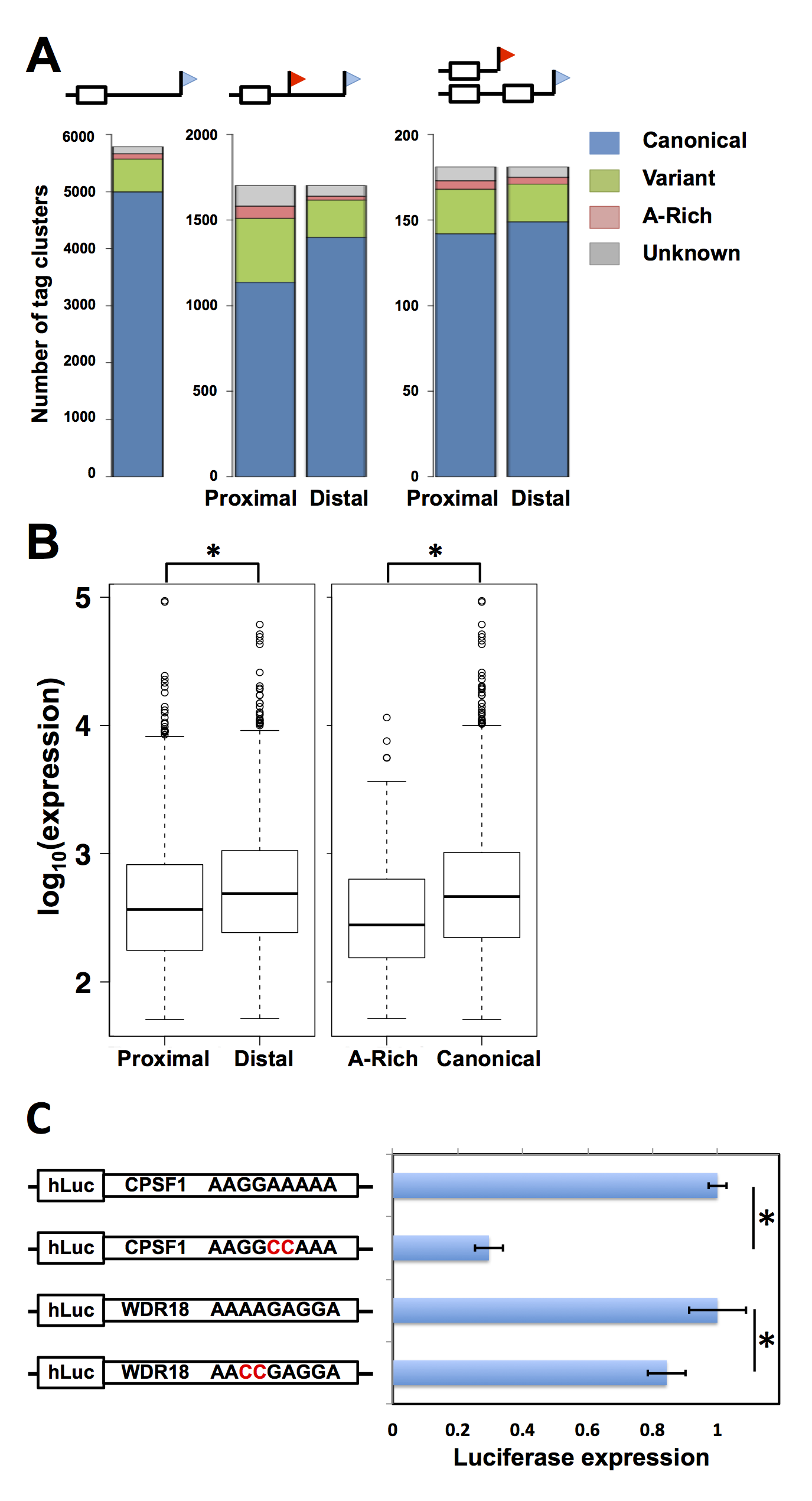

Supplement: Figure S3 — A-rich stretches as non-canonical polyadenylation signals. (A) At top, cartoons are schematics of alternative polyadenylation with symbols as in Figure 1B. Below, analysis of the inferred polyadenylation signals for each group of 3′-end RNA-seq reads defined as a transcript end (tag cluster; Materials and Methods). Each panel reports categorization of 3′ transcript forms with respect to the presence of polyadenylation sequence motifs upstream of inferred cleavage positions, for the set of genes with the indicated patterns of alternative polyadenylation. Canonical, A(A/U)UAAA; variant, one of 10 variants of the canonical motif, taken from [30]; A-rich, a match to the A-rich non-canonical polyadenylation motif (Materials and Methods); unknown, no match to any polyadenylation motif. “Proximal” and “Distal” represent polyadenylation signals upstream of proximal and distal transcript end forms, respectively. (B) Each panel represents analysis of the set of genes with evidence for alternative polyadenylation (class II or III; see Figure 1B in main text). Each column represents the distribution of 3′-end RNA-seq read counts, across all replicates and samples, in a set of transcript forms defined by the position of the transcript end in the 3′ untranslated region (left) or polyadenylation signal motif upstream of the cleavage site (right). For each distribution, the median is reported as a thick horizontal line, the 25% quantile is shown as a box, and the extremes are shown as thin horizontal bars. Asterisks represent comparisons significant by a two-sided Wilcoxon test at p<4×10^−6^. (C) Each row represents luciferase activity in HEK293T cells transfected with one reporter. Reporter schematics are at left; black nucleotides indicate inferred A-rich non-canonical polyadenylation signals in the human reference genome, and red nucleotides indicate mutated sites. Asterisks represent results significant at p<0.05 by a two-sided Wilcoxon test comparing the ratio of abundances betw [file pgen.1002882.s003.tiff]

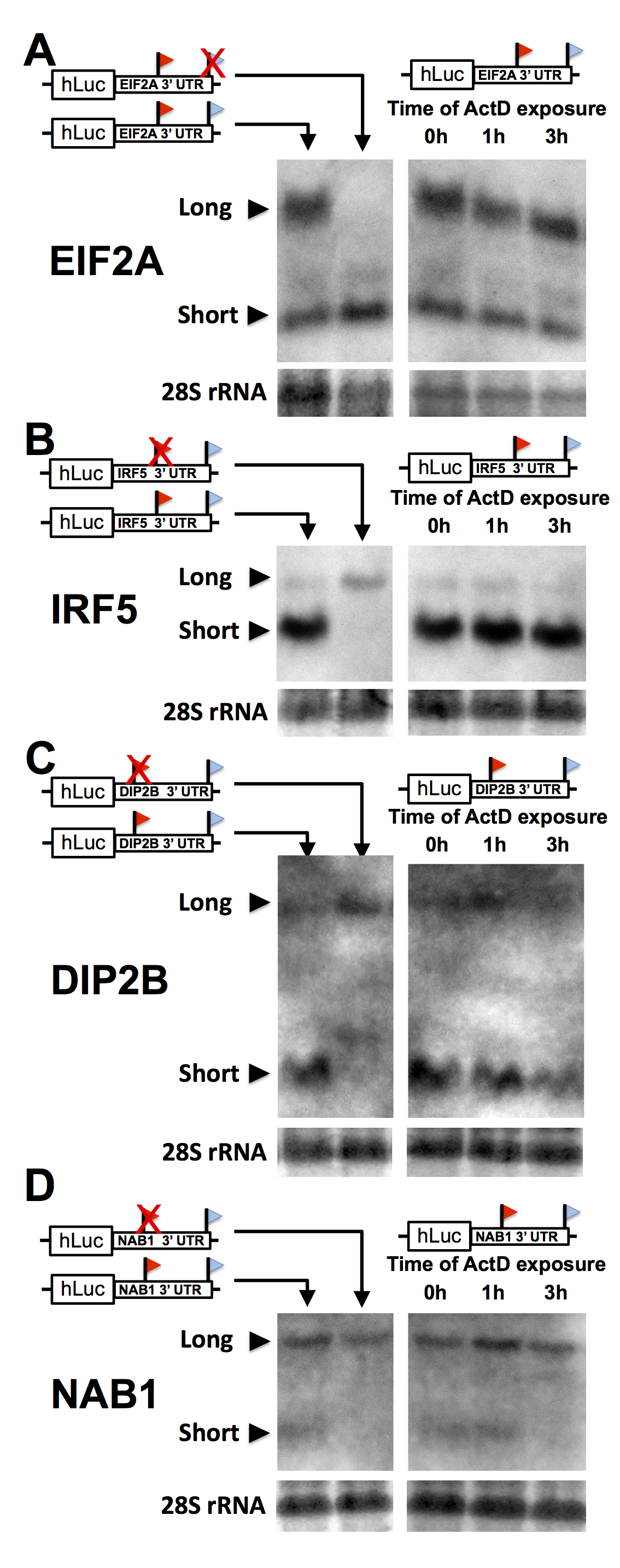

Supplement: Figure S4 — Variation in 3′ RNA length form usage assayed by Northern blot. Each row reports abundance of 3′ RNA length forms for one gene at which 3′ end usage varied across human B-lymphoblastoid cell lines from genetically distinct individuals (Figure 4). Cartoons at top represent luciferase reporters (hLuc) incorporating the complete 3′ untranslated region of the indicated gene, using a human haplotype producing both short and long 3′ forms (flags) or one bearing a naturally occurring allele that eliminates a canonical polyadenylation signal at one of the two termination sites (red X). For each experiment, a reporter was transfected into HEK293T cells which were then treated with actinomycin D for the indicated length of time (right panels) or untreated (left panels). In each panel, top images report intensities of hybridization of a colorimetric probe complementary to the reporter, and bottom images report intensities of 28S ribosomal RNA by SYBR Gold stain as a loading control. (A) EIF2A, (B) IRF5, (C) DIP2B, (D) NAB1. (TIFF) [file pgen.1002882.s004.tiff]

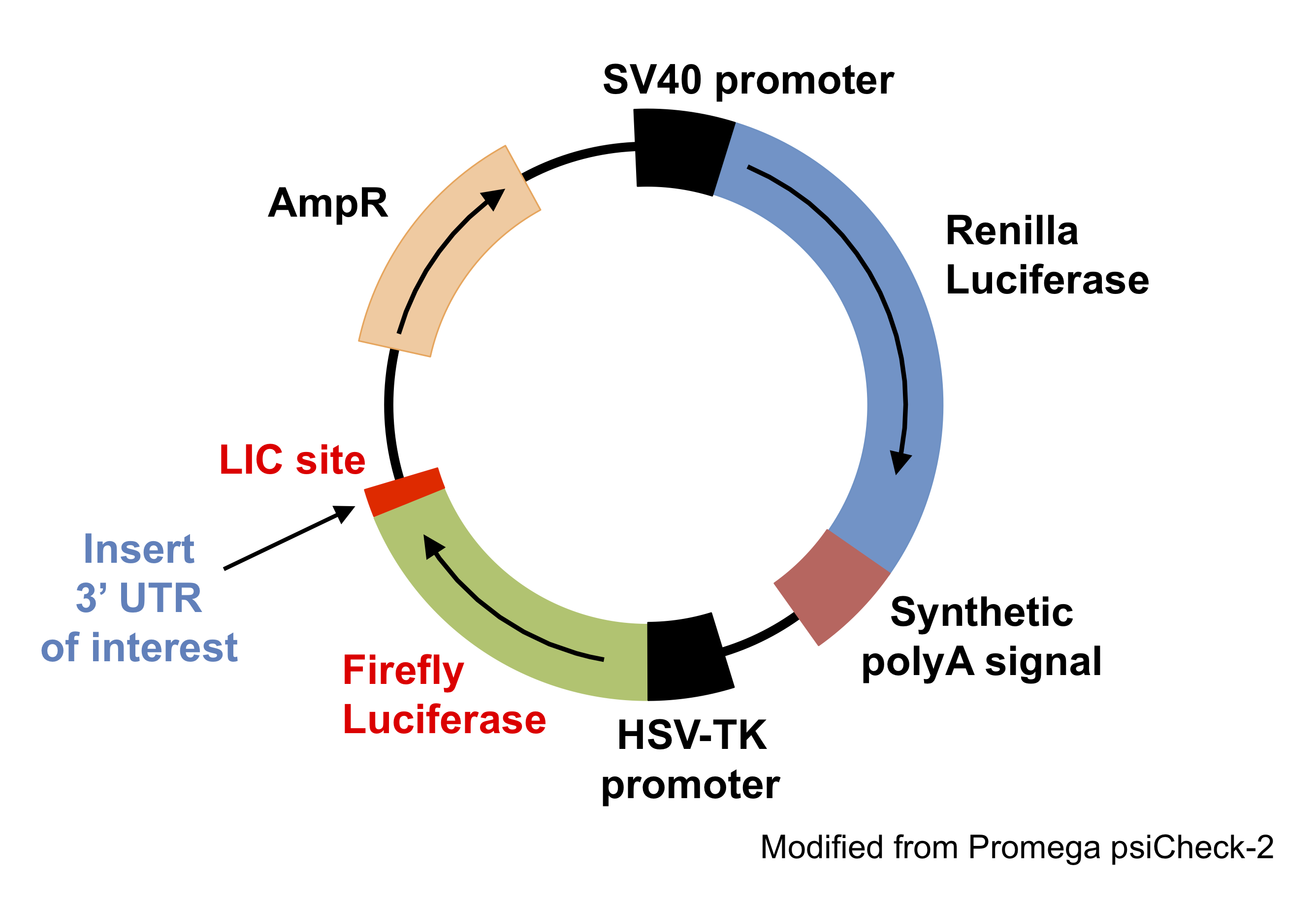

Supplement: Figure S5 — Schematic of cloning and reporter vector pOKY001. LIC, ligation independent cloning. (TIFF) [file pgen.1002882.s005.tiff]
